# Abiraterone or Enzalutamide for Patients With Metastatic Castration-Resistant Prostate Cancer

**DOI:** 10.1001/jamanetworkopen.2024.28444

**Published:** 2024-08-16

**Authors:** Jennifer La, Lin Wang, June K. Corrigan, Deborah Lang, Michelle H. Lee, Nhan V. Do, Mary T. Brophy, Channing J. Paller, Nathanael R. Fillmore

**Affiliations:** 1 VA Cooperative Studies Program, VA Boston Healthcare System, Boston, Massachusetts; 2 Department of Medicine, Harvard Medical School, Boston, Massachusetts; 3 Department of Epidemiology, Johns Hopkins Bloomberg School of Public Health, Baltimore, Maryland; 4 Department of Dermatology, Boston University Chobanian & Avedisian School of Medicine, Boston, Massachusetts; 5 Department of Medical Oncology, Mass General Cancer Center, Massachusetts General Hospital, Boston; 6 Section of General Internal Medicine, Boston University Chobanian & Avedisian School of Medicine, Boston, Massachusetts; 7 Section of Hematology & Medical Oncology, Boston University Chobanian & Avedisian School of Medicine, Boston, Massachusetts; 8 Department of Oncology, Johns Hopkins University School of Medicine, Baltimore, Maryland; 9 Department of Medical Oncology, Dana-Farber Cancer Institute, Boston, Massachusetts

## Abstract

**Importance:**

Abiraterone acetate and enzalutamide are recommended as preferred treatments for metastatic castration-resistant prostate cancer (mCRPC), but differences in their relative efficacy are unclear due to a lack of head-to-head clinical trials. Clear guidance is needed for making informed mCRPC therapeutic choices.

**Objective:**

To compare clinical outcomes in patients with mCRPC treated with abiraterone acetate or enzalutamide.

**Design, Setting, and Participants:**

This retrospective, multicenter cohort study included patients with mCRPC in the US Department of Veterans Affairs health care system who initiated treatment with abiraterone acetate or enzalutamide between January 1, 2014, and October 30, 2022.

**Exposures:**

Abiraterone acetate or enzalutamide.

**Main Outcomes and Measures:**

The study used inverse probability of treatment weighting to balance baseline characteristics between patients initiating abiraterone acetate or enzalutamide and evaluated restricted mean survival time (RMST) differences in overall survival (OS), prostate cancer–specific survival (PCS), time to next treatment switching or death (TTS), and time to prostate-specific antigen (PSA) response (TTR) at different time points after treatment initiation.

**Results:**

The study included 5779 patients (median age, 74.42 years [IQR, 68.94-82.14 years]). Median follow-up was between 38 and 60 months. Patients initiating enzalutamide on average had longer OS than those initiating abiraterone acetate, with RMSTs of 24.29 months (95% CI, 23.58-24.99 months) and 23.38 months (95% CI, 22.85-23.92 months), respectively, and a difference in RMST of 0.90 months (95% CI, 0.02-1.79 months) at 4 years. Similarly, TTS and TTR were improved in patients initiating enzalutamide, with an RMST at 4 years of 1.95 months (95% CI, 0.92-2.99 months) longer for TTS and 3.57 months (95% CI, 1.76-5.38 months) shorter for TTR. For PCS, the RMST at 2 years was 0.48 months (95% CI, 0.01-0.95 months) longer. An examination of subgroups identified that enzalutamide initiation was associated with longer RMST in OS among patients without prior docetaxel treatment (1.14 months; 95% CI, 0.19-2.10 months) and in those with PSA doubling time of 3 months or longer (2.23 months; 95% CI, 0.81-3.66 months) but not among patients with prior docetaxel (−0.25 months; 95% CI, −2.59 to 2.09 months) or with PSA doubling time of less than 3 months (0.05 months; 95% CI, −1.05 to 1.15 months).

**Conclusions and Relevance:**

In this cohort study of patients with mCRPC, initiation of enzalutamide was associated with small but statistically significant improvements in OS, PCS, TTS, and TTR compared with initiation of abiraterone acetate. The improvements were more prominent in short-term outcomes, including TTS and TTR, and in patient subgroups without prior docetaxel or with PSA doubling time longer than 3 months.

## Key Points

**Question:**

Are there differences in outcomes for patients initiating treatment with abiraterone acetate vs enzalutamide for metastatic castration-resistant prostate cancer (mCRPC)?

**Findings:**

In this cohort study of 5779 patients with mCRPC from the US Department of Veterans Affairs health care system from 2014 to 2022, initiation of enzalutamide was associated with small but statistically significant improvements in survival times compared with initiation of abiraterone acetate, with more prominent improvements in short-term outcomes and in patient subgroups with less aggressive prostate cancer.

**Meaning:**

These findings may provide guidance for treatment strategies for patients with mCRPC.

## Teaser

This cohort study uses data from the US Department of Veterans Affairs to compare clinical outcomes of treatment initiation of abiraterone acetate vs enzalutamide in patients with metastatic castration-resistant prostate cancer.

## Introduction

Abiraterone acetate and enzalutamide are compounds that inhibit the androgen receptor (AR) signaling pathway and are often used as first-line treatment for metastatic castration-resistant prostate cancer (mCRPC) instead of more toxic agents such as docetaxel.^1,2,3,4,5^ While abiraterone acetate and enzalutamide are different in their mechanisms of action, toxicities, and costs, both compounds are recommended for mCRPC.^6,7,8,9,10^ Nevertheless, while both compounds are effective and widely used, their relative benefits and risks are still not fully understood. Each agent has been extensively studied in separate clinical trials including the pivotal trials leading to approval,^11,12,13,14^ but to our knowledge, they have not been compared in large-scale, head-to-head trials. Observational studies comparing the 2 agents have been limited as to sample size, study design, length of follow-up, lack of information on key variables such as castration resistance, or a combination of these factors.^15,16,17,18,19,20^

The purpose of the present study was to compare clinical outcomes in patients initiating treatment for mCRPC with abiraterone acetate or enzalutamide from 2014 to 2022 using a large, retrospective cohort in the national US Department of Veterans Affairs (VA) health care system. We aimed to quantify the clinical outcomes of these 2 treatments using rigorous methods in a large cohort with robust follow-up and carefully defined data elements.

## Methods

### Study Design, Data Sources, and Participants

This retrospective cohort study compared outcomes in patients with mCRPC in the VA health care system who initiated treatment with abiraterone acetate vs enzalutamide. Data were obtained from the VA Corporate Data Warehouse (CDW), which collates administrative and electronic health record data from VA facilities throughout the US.^21^ This study was approved by the VA Boston Healthcare System Research and Development Committee as an exempt study prior to data collection and analysis, with a waiver of informed consent per the Common Rule due to use of existing data. This report followed the Strengthening the Reporting of Observational Studies in Epidemiology (STROBE) reporting guideline for cohort studies.^22^

The study included patients with mCRPC initiating treatment with abiraterone acetate or enzalutamide between January 1, 2014, and October 30, 2022. Treatment initiation (the index date) was required to be on or after January 1, 2014, since there was limited uptake of enzalutamide before this date. Treatments given in inpatient and outpatient settings at the VA as well as claims for outside care paid for by the VA were captured. Castration resistance was based on Prostate Cancer Clinical Trials Working Group 3 (PCWG3) criteria. Specifically, patients were considered to be castration resistant if they underwent orchiectomy (codes listed in the eMethods in Supplement 1) or androgen-deprivation therapy (treatments listed in the eMethods in Supplement 1) and had prostate-specific antigen (PSA) progression, defined as a PSA level increase greater than 25%, with measurements at least 3 weeks apart. Metastatic status was obtained using a 2-step process. First, a previously published and validated natural language processing algorithm was used to identify patients with potentially metastatic disease based on clinical notes and radiology reports and to obtain the date on which metastatic disease was first recorded.^23^ Second, patients’ medical records were manually reviewed by a medical oncologist (M.H.L.) or a board-certified radiologist to confirm metastatic status. Patients without castration-resistant and metastatic status before or within 30 days after the initial treatment with abiraterone acetate or enzalutamide were excluded. Patients were also excluded if they lacked demographic data, follow-up data, or a baseline PSA value within 365 days prior to the index date.

### Outcomes and Covariates

Four time-to-event outcomes were evaluated: overall survival (OS), prostate cancer–specific survival (PCS), time to PSA response (TTR), and time to treatment switching or death (TTS). We defined OS as the time from the index date to death from any cause as recorded in the VA CDW. Patients were censored on October 31, 2022 (the end of the study period). Mortality data at the VA are highly accurate and complete,^24^ so it was unnecessary to censor for loss to follow-up. We defined PCS as the time from the index date to death from prostate cancer, as recorded on the death certificate from the National Death Index and linked to VA patient identifiers using the VA Mortality Data Repository. Patients were censored on the date they died from another cause or on December 31, 2019, which at the time of analysis was the date the most recent data were available in the Mortality Data Repository. We defined TTR as the time from the index date to a PSA level decline of at least 50% based on the PCWG3 definition (details are in the eMethods in Supplement 1).^25^ Since it is possible that patients could start treatment at the VA and later switch to an outside institution, which would lead to missing PSA response data, patients were censored at the end of the study period, the date of treatment switching or death, or the first break in continuous follow-up after the index date, defined as the end of a 90-day gap without any clinical encounter at the VA or the last PSA laboratory test result. We defined TTS as the time from the index date to a new prostate cancer treatment (listed in the eMethods in Supplement 1) after the initiation of abiraterone acetate or enzalutamide or as the time of death, whichever occurred first. Patients were censored at the end of the study period or at the first break in continuous follow-up after the index date.

Covariates were defined based on data recorded prior to the index date. Age and self-reported race and ethnicity were ascertained using structured data in the VA CDW. Race and ethnicity were included because they are potential confounders, and we sought to evaluate outcomes in subgroups defined by race and ethnicity; categories were Hispanic, non-Hispanic Black (hereafter, Black), non-Hispanic White (hereafter, White), and other or unknown race and ethnicity (included American Indian or Alaska Native, Asian, Native Hawaiian or Other Pacific Islander, declined to answer, and unknown by patient). Frailty was measured using the VA Frailty Index,^26^ and individual comorbidities were measured using definitions from the Centers for Medicare & Medicaid Services Chronic Conditions Data Warehouse^27^ based on both diagnosis and procedure codes recorded in the 3 years prior to the index date. Prior treatment information and laboratory test results were obtained from structured data. Detailed definitions, including details of race and ethnicity categories, are in the eMethods in Supplement 1.

### Statistical Analysis

Inverse probability of treatment weighting (IPTW) was used to control for potential confounders, including age, race and ethnicity, comorbidities, frailty, treatment initiation year, prior treatment, baseline PSA level, and PSA doubling time. The estimated probability (ie, propensity score) of a patient starting enzalutamide vs abiraterone acetate given the patient’s characteristics at baseline was determined using multivariable logistic regression that included these potential confounders as covariates. We did not include non–log-linear associations or covariate interactions because the treatment groups were well balanced after IPTW without including these. The distribution of propensity scores was examined for overlap between the 2 treatment groups. The propensity scores were used to calculate stabilized weights for IPTW.^28^ Stabilized weights were examined for extreme outliers. After weighting, balance was inspected by tabulating weighted patient characteristics within the treatment groups and examining the standardized mean difference (SMD) across groups, where SMD less than 0.1 conventionally indicates good balance.^29^

After IPTW, Kaplan-Meier analysis was used to estimate the survival function for each outcome stratified by treatment type. Cox proportional hazards regression models were not used due to observed violation of the assumption of proportional hazards through graphical examination of the hazard ratio over time and the global Schoenfeld residuals test. An estimation was made of restricted mean survival time (RMST), which is defined as the area under the survival curve up to specific time points, following previously published methods.^30^ The RMST differences were used to compare patients initiating enzalutamide with those initiating abiraterone acetate. Analyses were also conducted in preplanned subgroups defined by PSA doubling time, prior use of docetaxel, age, and race and ethnicity. For each subgroup analysis, the propensity score was reestimated to ensure balance. Median follow-up was determined using the reverse Kaplan-Meier estimator. All analyses were conducted using R, version 4.1.2 (R Project for Statistical Computing).

## Results

### Cohort Characteristics

**Figure 1.**
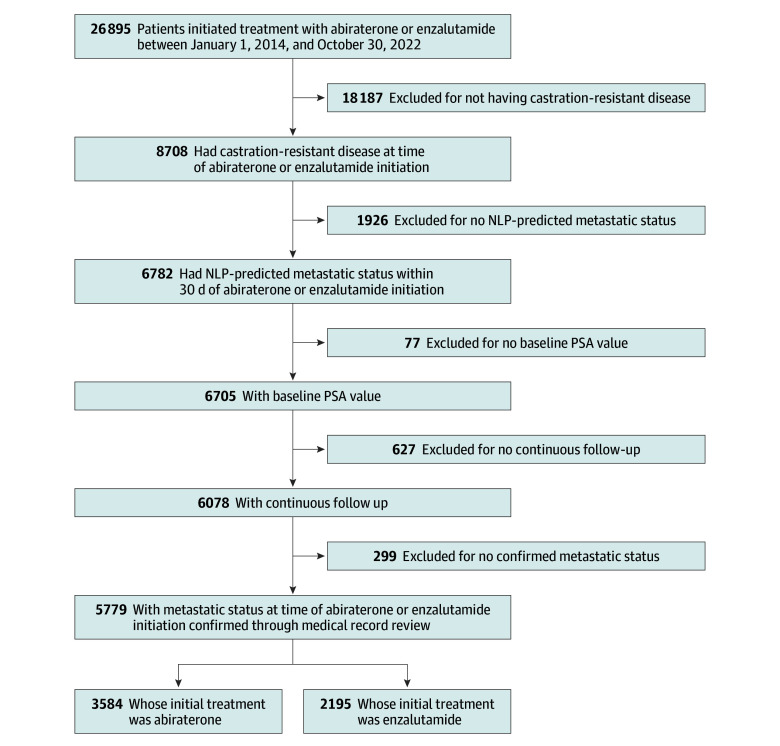
Flow Diagram Depicting Selection of Study Cohort NLP indicates natural language processing; PSA, prostate-specific antigen.

The inclusion criteria were met by 5779 patients (Figure 1) with a median age of 74.42 years (IQR, 68.94-82.14 years); 1583 (27.4%) were Black; 313 (5.4%), Hispanic; 3525 (61.0%), White; and 358 (6.2%), other or unknown race and ethnicity. The Table summarizes patient characteristics before and after IPTW. Prior to reweighting, patients initially treated with abiraterone acetate were younger, more likely to have received prior treatment with docetaxel and bisphosphonate, less likely to have had prior radiation therapy, and less likely to have comorbidities (including diabetes, heart failure, chronic kidney disease, Alzheimer disease, and peripheral vascular disease). They also had a higher median PSA level at baseline compared with patients initially treated with enzalutamide. After reweighting, patient characteristics were well balanced (SMD<0.1) (Table and eFigure 1 in Supplement 1).^29^ There was no evidence of nonpositivity or misspecification of the propensity score model based on an examination of score distributions (eFigure 2 in Supplement 1). The mean stabilized weight was 1.0, and there were no extreme weights (minimum stabilized weight, 0.4; maximum, 3.1) (eFigure 3 in Supplement 1). For OS, median follow-up was 60 months (IQR, 57-64 months) for abiraterone acetate and 55 months (IQR, 50-57 months) for enzalutamide. For PCS, median follow-up was 41 months (IQR, 39-42 months) for abiraterone acetate and 38 months (IQR, 37-39 months) for enzalutamide. The global Schoenfeld residuals test and plots of the hazard ratio over time showed evidence of violation of the assumption of proportional hazards (eFigures 4-13 in Supplement 1).

**Table.** Patient Characteristics Before and After Inverse Probability of Treatment Weighting

Characteristic	Unweighted^a^				Weighted^a^			
	Overall (N = 5779)	Abiraterone (n = 3584)	Enzalutamide (n = 2195)	SMD	Overall (n = 5776)	Abiraterone (n = 3584)	Enzalutamide (n = 2192)	SMD
Age, median (IQR), y	74.42 (68.94-82.14)	74.09 (68.70-81.52)	75.03 (69.35-82.88)	0.102	74.40 (68.94-82.14)	74.41 (69.04-81.98)	74.40 (68.79-82.45)	0.003
Race and ethnicity								
Hispanic	313 (5.4)	172 (4.8)	141 (6.4)	0.089	311.9 (5.4)	193.8 (5.4)	118.1 (5.4)	0.009
Non-Hispanic Black	1583 (27.4)	956 (26.7)	627 (28.6)		3510.1 (60.8)	2182.8 (60.9)	1327.3 (60.5)	
Non-Hispanic White	3525 (61.0)	2232 (62.3)	1293 (58.9)		1591.7 (27.6)	982.4 (27.4)	609.3 (27.8)	
Other or unknown^b^	358 (6.2)	224 (6.2)	134 (6.1)		362.7 (6.3)	225.0 (6.3)	137.7 (6.3)	
Year of initial treatment								
2014	859 (14.9)	700 (19.5)	159 (7.2)	0.404	855.8 (14.8)	532.6 (14.9)	323.2 (14.7)	0.004
2015	791 (13.7)	488 (13.6)	303 (13.8)		792.4 (13.7)	491.2 (13.7)	301.2 (13.7)	
2016	782 (13.5)	449 (12.5)	333 (15.2)		780.2 (13.5)	484.1 (13.5)	296.1 (13.5)	
2017	806 (13.9)	476 (13.3)	330 (15.0)		808.7 (14.0)	501.1 (14.0)	307.5 (14.0)	
2018	731 (12.6)	443 (12.4)	288 (13.1)		735.5 (12.7)	455.8 (12.7)	279.7 (12.8)	
2019	616 (10.7)	319 (8.9)	297 (13.5)		616.1 (10.7)	381.8 (10.7)	234.3 (10.7)	
2020	471 (8.2)	250 (7.0)	221 (10.1)		468.4 (8.1)	290.5 (8.1)	177.9 (8.1)	
2021	415 (7.2)	246 (6.9)	169 (7.7)		411.6 (7.1)	255.9 (7.1)	155.6 (7.1)	
2022	308 (5.3)	213 (5.9)	95 (4.3)		307.9 (5.3)	191.0 (5.3)	116.8 (5.3)	
Prior treatment								
Docetaxel	775 (13.4)	525 (14.6)	250 (11.4)	0.097	785.2 (13.6)	483.6 (13.5)	301.6 (13.8)	0.008
Radiation therapy	526 (9.1)	312 (8.7)	214 (9.7)	0.036	524.3 (9.1)	325.7 (9.1)	198.6 (9.1)	0.001
Prostatectomy	469 (8.1)	297 (8.3)	172 (7.8)	0.017	463.4 (8.0)	289.2 (8.1)	174.3 (7.9)	0.004
Bisphosphonate	2161 (37.4)	1393 (38.9)	768 (35.0)	0.080	2163.8 (37.5)	1339.4 (37.4)	824.4 (37.6)	0.005
Baseline PSA level, median (IQR), ng/mL	30.00 (11.20-91.83)	30.52 (11.25-93.83)	29.10 (10.96-87.62)	0.020	30.00 (11.17-91.47)	29.73 (11.06-92.18)	30.02 (11.40-90.20)	0.003
PSA doubling time, median (IQR), mo	2.59 (1.50-4.58)	2.52 (1.46-4.48)	2.68 (1.58-4.77)	0.031	2.50 (1.53-4.48)	2.45 (1.51-4.45)	2.56 (1.58-4.60)	0.019
Comorbidities								
Acute myocardial infarction	185 (3.2)	111 (3.1)	74 (3.4)	0.016	180.2 (3.1)	113.3 (3.2)	66.8 (3.0)	0.007
Atrial fibrillation	809 (14.0)	483 (13.5)	326 (14.9)	0.039	821.6 (14.2)	506.2 (14.1)	315.4 (14.4)	0.007
Cardiac arrhythmia	1750 (30.3)	1053 (29.4)	697 (31.8)	0.052	1761.3 (30.5)	1088.0 (30.4)	673.3 (30.7)	0.008
Cardiovascular disease	3260 (56.4)	1970 (55.0)	1290 (58.8)	0.077	3271.9 (56.6)	2024.1 (56.5)	1247.9 (56.9)	0.009
Complicated hypertension	1063 (18.4)	620 (17.3)	443 (20.2)	0.074	1073.9 (18.6)	661.9 (18.5)	412.0 (18.8)	0.008
Congestive heart failure	907 (15.7)	493 (13.8)	414 (18.9)	0.139	915.9 (15.9)	564.9 (15.8)	351.0 (16.0)	0.007
Heart failure	834 (14.4)	452 (12.6)	382 (17.4)	0.134	840.2 (14.5)	519.0 (14.5)	321.2 (14.7)	0.005
Peripheral vascular disease	1105 (19.1)	627 (17.5)	478 (21.8)	0.108	1098.9 (19.0)	682.9 (19.1)	416.0 (19.0)	0.002
Stroke or transient ischemic attack	502 (8.7)	318 (8.9)	184 (8.4)	0.017	504.3 (8.7)	311.2 (8.7)	193.1 (8.8)	0.004
Valvular disease	488 (8.4)	285 (8.0)	203 (9.2)	0.046	494.9 (8.6)	305.9 (8.5)	189.0 (8.6)	0.003
Alzheimer disease	591 (10.2)	339 (9.5)	252 (11.5)	0.066	590.6 (10.2)	365.9 (10.2)	224.7 (10.2)	0.001
Anemia	2463 (42.6)	1497 (41.8)	966 (44.0)	0.045	2468.4 (42.7)	1529.0 (42.7)	939.3 (42.8)	0.004
Cerebrovascular disease	792 (13.7)	482 (13.4)	310 (14.1)	0.020	794.9 (13.8)	490.9 (13.7)	304.0 (13.9)	0.005
Chronic kidney disease	2301 (39.8)	1345 (37.5)	956 (43.6)	0.123	2312.1 (40.0)	1429.2 (39.9)	882.9 (40.3)	0.008
Diabetes	2170 (37.5)	1245 (34.7)	925 (42.1)	0.153	2183.0 (37.8)	1349.1 (37.6)	833.9 (38.0)	0.008
Liver disease	445 (7.7)	261 (7.3)	184 (8.4)	0.041	446.2 (7.7)	276.7 (7.7)	169.5 (7.7)	<0.001
Viral hepatitis	250 (4.3)	153 (4.3)	97 (4.4)	0.007	253.2 (4.4)	156.5 (4.4)	96.7 (4.4)	0.002
VA Frailty Index, median (IQR)^c^	0.23 (0.16-0.32)	0.23 (0.16-0.32)	0.23 (0.16-0.32)	0.139	0.23 (0.16-0.32)	0.23 (0.16-0.32)	0.23 (0.16-0.32)	0.008
Hemoglobin level, median (IQR), g/dL	12.20 (10.90-13.30)	12.30 (11.00-13.40)	12.10 (10.80-13.30)	0.054	12.20 (10.90-13.30)	12.30 (11.00-13.30)	12.10 (10.82-13.30)	0.022
LDH, median (IQR)	192.00 (161.75-250.25)	193.00 (162.00-254.00)	190.00 (160.00-246.00)	0.080	191.00 (161.00-250.00)	192.00 (162.00-253.87)	189.00 (158.00-239.88)	0.074

Abbreviations: LDH, lactate dehydrogenase; PSA, prostate-specific antigen; SMD, standardized mean difference; VA, Veterans Affairs.

SI conversion factors: To convert PSA to micrograms per liter, multiply by 1.0; hemoglobin to grams per liter, multiply by 10.0.

^a^ Data are presented as number (percentage) of patients unless otherwise indicated.

^b^ Includes American Indian or Alaska Native, Asian, Native Hawaiian or Other Pacific Islander, declined to answer, and unknown by patient.

^c^ On a scale of 0 to 1, with higher scores indicating increased frailty.

### Short-Term Outcomes of Enzalutamide in the Full Cohort

**Figure 2.**
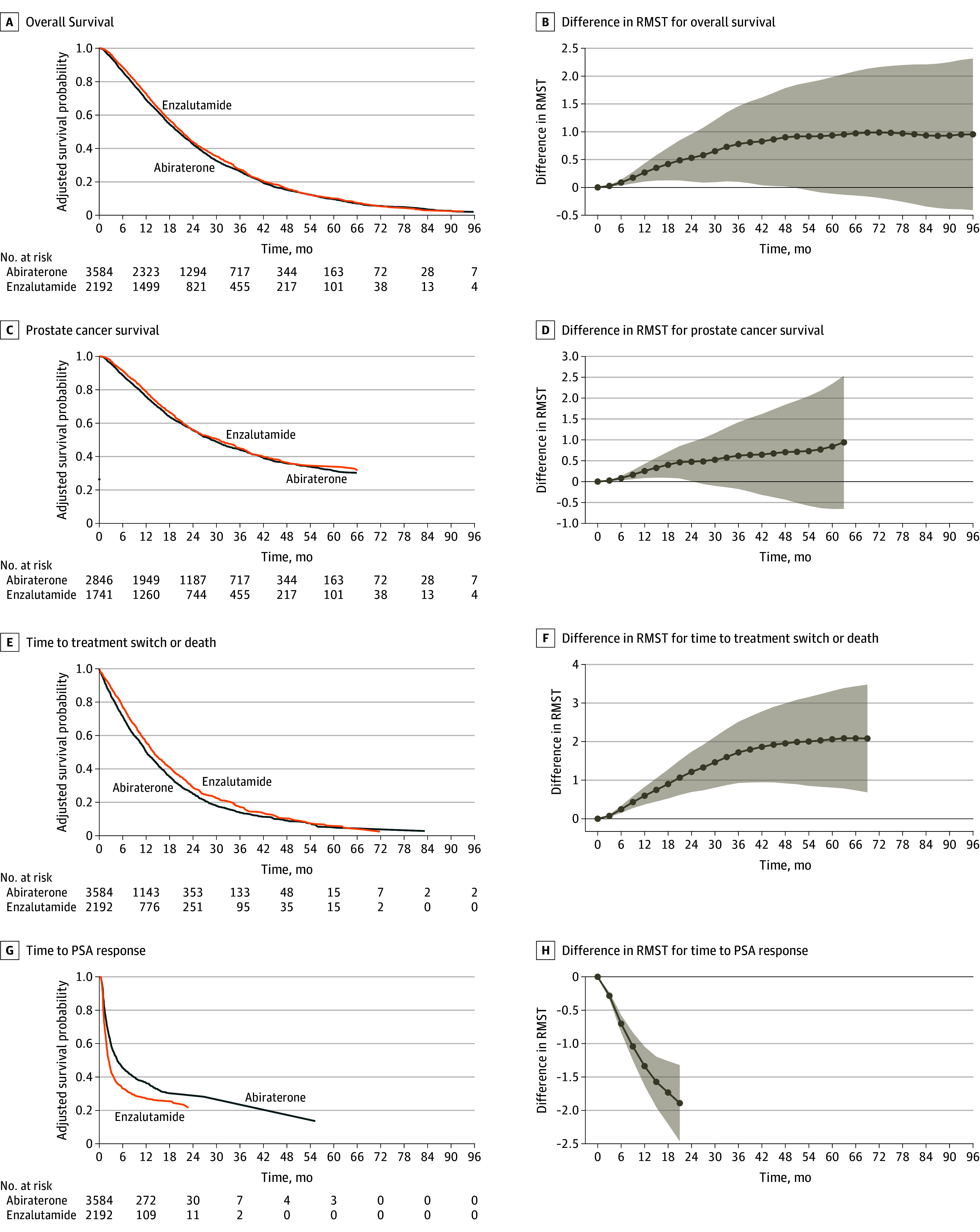
Outcomes in Patients Initially Treated With Abiraterone Acetate vs Enzalutamide After Inverse Probability of Treatment Weighting Kaplan-Meier plots and the difference in restricted mean survival time (RMST) at each time point (3-month increments) are shown. The RMST at a given time point measures the mean survival censoring at the time point and is equal to the area under the Kaplan-Meier plot up to the time point. PSA indicates prostate-specific antigen.

Enzalutamide was associated with meaningful improvements in short-term outcomes (TTS and TTR) after treatment initiation compared with abiraterone acetate (Figure 2C and D), but these improvements were more subtle in long-term outcomes (OS and PCS) (Figure 2A and B). The eTable in Supplement 1 reports the RMST for patients initially treated with enzalutamide or abiraterone acetate at 1 to 4 years after treatment initiation for all outcomes. Among patients initially treated with enzalutamide, the RMST for OS was 0.27 months (95% CI, 0.11-0.43 months) longer at 1 year after treatment initiation compared with those initially treated with abiraterone acetate, rising to 0.53 months (95% CI, 0.10-0.96 months) longer at 2 years, 0.78 months (95% CI, 0.10-1.46 months) longer at 3 years, and 0.90 months (95% CI, 0.02-1.79 months) longer at 4 years (Figure 2A). In terms of OS, at 4 years, patients who initiated enzalutamide had an RMST of 24.29 months (95% CI, 23.58-24.99 months) and those who initiated abiraterone acetate had an RMST of 23.38 months (95% CI, 22.85-23.92 months). For PCS (Figure 2B), compared with those initially treated with abiraterone acetate, patients initially treated with enzalutamide had 0.25 months (95% CI, 0.09-0.42 months) longer RMST at 1 year and 0.48 months (95% CI, 0.01-0.95 months) longer RMST at 2 years after treatment initiation, but there was no significant difference in the RMST at 3 years (0.62 months; 95% CI, −0.18 to 1.42 months) or 4 years (0.71 months; 95% CI, −0.43 to 1.84 months). For TTS, the differences were also in favor of enzalutamide (Figure 2C). The RMST was 0.60 months (95% CI, 0.37-0.82 months) longer at 1 year after treatment initiation for patients initially treated with enzalutamide compared with those initiating abiraterone acetate, rising to 1.21 months (95% CI, 0.69-1.74 months) longer at 2 years, 1.72 months (95% CI, 0.93-2.51 months) longer at 3 years, and 1.95 months (95% CI, 0.92-2.99 months) longer at 4 years. In addition, TTR was shorter among patients initially treated with enzalutamide compared with those initiating abiraterone acetate (Figure 2D). The RMST was 1.34 months (95% CI, 1.05-1.63 months) shorter at 1 year after treatment initiation, 2.10 months (95% CI, 1.42-2.78 months) shorter at 2 years, 2.93 months (95% CI, 1.67-4.19 months) shorter at 3 years, and 3.57 months (95% CI, 1.76-5.38 months) shorter at 48 months after treatment initiation.

### Outcomes of Enzalutamide Among Patients With Less Progressive Prostate Cancer

**Figure 3.**
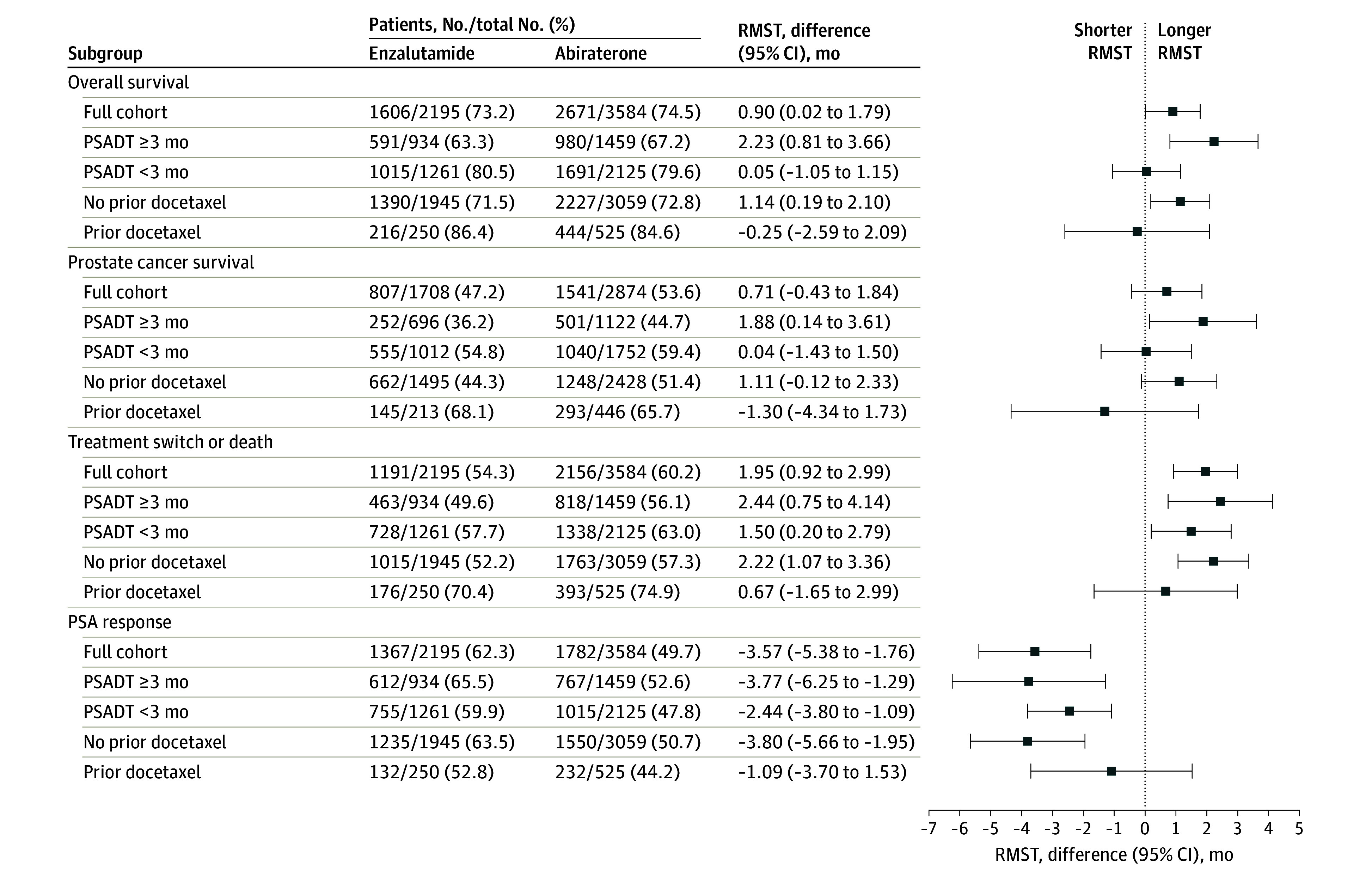
Differences in Restricted Mean Survival Time (RMST) at 4 Years After Treatment Initiation for the Full Cohort and for Subgroups Defined by Prostate-Specific Antigen Doubling Time (PSADT) and Prior Receipt of Docetaxel

Among patients with PSA doubling time of 3 months or longer or without prior docetaxel treatment, there was a statistically significant improvement in all 4 outcomes (OS, PCS, TTS, and TTR) at early time points and for OS, TTS, and TTR at 4 years (Figure 3 and eFigures 14 and 15 in Supplement 1). For example, enzalutamide initiation was associated with a longer RMST in OS at 4 years in patients without prior docetaxel treatment (RMST difference, 1.14 months; 95% CI, 0.19-2.10 months) or with PSA doubling time of 3 months or longer (RMST difference, 2.23 months; 95% CI, 0.81-3.66 months). Differences between treatments were more subtle in patients with more aggressive prostate cancer. There were no statistically significant differences between abiraterone acetate and enzalutamide for OS and PCS in patients with a PSA doubling time of less than 3 months (RMST difference in OS at 4 years, 0.05 months; 95% CI, −1.05 to 1.15 months) (eFigure 16 in Supplement 1) or with prior docetaxel treatment (RMST difference in OS at 4 years, −0.25 months; 95% CI, −2.59 to 2.09 months) (eFigure 17 in Supplement 1). Results in subgroups defined by age and by race and ethnicity were generally similar to results in the overall cohort (eFigures 18-22 in Supplement 1).

## Discussion

Using the largest integrated health care system in the US, this study compared outcomes in 5779 patients initiating abiraterone acetate or enzalutamide for the treatment of mCRPC with rigorous methods and current nationwide patient data. The study showed that initial enzalutamide treatment, in general, was associated with more favorable outcomes than initial abiraterone acetate treatment, although the differences were small. The improvements were more prominent in short-term outcomes, including TTS and TTR, and in patient subgroups with less aggressive prostate cancer (without prior docetaxel or with PSA doubling time ≥3 months).

The different mechanism of action might explain why enzalutamide outperformed abiraterone acetate in some subgroups. Abiraterone acetate inhibits the CYP17A enzyme, a critical component of androgen synthesis. Abiraterone acetate intensifies testosterone suppression and works upstream in the pathway, whereas enzalutamide blocks multiple downstream events, including AR nuclear translocation and transcription. One possible reason for differential responses between treatment regimens is that in some prostate cancers, AR is overactive or mutated independent of abiraterone acetate–targeted upstream events.^31,32,33,34^ The AR sequence variant may be resistant to upstream therapeutic targeting but still sensitive to enzalutamide.^35^ Of note, using both enzalutamide and abiraterone acetate together as a first-line combination treatment did not yield significant improvements in OS.^36^

This study complements existing data from meta-analyses of clinical trials and studies in retrospective cohorts.^37,38,39,40,41^ To our knowledge, there have not been head-to-head clinical trials comparing abiraterone acetate and enzalutamide. Meta-analyses are limited by a lack of head-to-head trials and the strict inclusion criteria and relatively short follow-up of existing trials.^37^ For example, clinical trials of abiraterone acetate and enzalutamide for mCRPC^11,12,42,43^ excluded patients with severe comorbidities, such as cardiovascular diseases, brain and/or visceral organ metastases, and other malignant neoplasms. Therefore, findings from trials and subsequent meta-analyses may not be generalizable to these patients. Previous meta-analyses suggested that enzalutamide outperformed abiraterone acetate among patients in mCRPC trials in radiographic progression-free survival,^38,39,40,41^ time to PSA progression,^38,40^ and PSA response rate.^38^ Our study also showed that long-term OS and PCS benefits were associated with initiating enzalutamide for mCRPC and supported previous findings among a broader patient population in a clinical setting.^38,39,40,41^

Compared with previous retrospective studies,^18,19,20,44,45^ this study provided more information on mCRPC treatment options by investigating a larger cohort with more recent data, observing follow-up of patients for a longer duration with a comprehensive set of outcomes, and applying more rigorous methods. Two previous studies conducted in Turkey^45^ and Spain^44^ had small sample sizes of 250 and 90 patients, respectively, and limited durations of follow-up (medians of 41 and 25 months). While Demirci and colleagues^45^ found significantly longer radiographic progression-free survival and OS in the enzalutamide group than in the abiraterone acetate group through regression analysis controlling for potential confounders, Cabetas and colleagues^44^ found no difference in OS but without confounder control. Two retrospective studies using data from the VA had large sample sizes (5822 and 3174 patients), and both adjusted for age, race, comorbidities, and prior treatments through regression.^18,19^ However, patients in these 2 studies were only followed up through 2017, with median durations of 18 or 23 months, and long-term outcomes could not be fully evaluated. Tagawa and colleagues^19^ focused on chemotherapy-naive patients with mCRPC only and found that enzalutamide was associated with 16% lower mortality than abiraterone acetate. Similarly, Schoen and colleagues^18^ found that enzalutamide was associated with decreased mortality compared with abiraterone acetate in both multivariable and propensity score–matched analysis. George and colleagues^20^ reported similar findings using Surveillance, Epidemiology, and End Results–Medicare data but only with patients initially treated from 2014 to 2017, only in chemotherapy-naive patients, and with a relatively short median follow-up of approximately 20 months. Our study reported results consistent with these earlier studies for shorter-term survival but added substantially longer follow-up, inclusion of individuals initiating treatment after 2017, evaluation of both chemotherapy-naive and non–chemotherapy-naive patients, and/or careful control of confounding through IPTW. Our study included 5779 patients for a median follow-up of 38 to 60 months. In addition to OS, we examined PCS, TTR, and TTS. This provided a holistic picture of disease trajectory and patient journey, with results suggesting the superiority of enzalutamide initiation over abiraterone acetate across different outcomes among patients with mCRPC.

### Limitations

Despite its strengths, our study has limitations. Although we adjusted for many potential confounders using IPTW, there are additional potential confounders that we were unable to include due to data availability, including diagnostic Gleason score, tumor volume, number and location of metastases, time since previous treatment, and duration of previous treatment. In addition, although we balanced the cohort across comorbidities, the US veteran population has an overall high burden of comorbidity, and results could differ in populations with less comorbidity.

## Conclusions

In this cohort study of patients with mCRPC, we found that initial enzalutamide treatment, in general, was associated with more favorable outcomes than initial abiraterone acetate treatment. The improvements were more prominent in short-term outcomes, including TTS and TTR, and in patient subgroups with less aggressive prostate cancer (without prior docetaxel treatment or with PSA doubling time ≥3 months). The findings of this large-scale observational study with robust follow-up and rigorous methods may provide guidance for making well-informed decisions about mCRPC treatment strategies.

## Notes

Supplement 1.
eMethods.
**eTable.** RMST and Difference in RMST Between Enzalutamide Initiators and Abiraterone Acetate Initiators at 12, 24, 36, and 48 Months After Index Date for OS, PCS, TTS, and TTR **eFigure 1.** Standardized Mean Differences of Variables Before Inverse Probability of Treatment Weighting and After Weighting **eFigure 2.** Distribution of the Propensity Score for Patients Initially Treated With Abiraterone and Enzalutamide in the Unweighted Dataset **eFigure 3.** Inverse Probability of Treatment Weights for Patients Initially Treated With Abiraterone and Enzalutamide in the Unweighted Dataset **eFigure 4**. Hazard Ratios Over Time in the Full Cohort for Patients Initially Treated With Abiraterone Acetate vs Enzalutamide After Inverse Probability of Treatment Weighting **eFigure 5**. Hazard Ratios Over Time Among Patients With PSA Doubling Time of 3 Months or More Initially Treated With Abiraterone Acetate vs Enzalutamide After Inverse Probability of Treatment Weighting **eFigure 6**. Hazard Ratios Over Time Among Patients With No Prior Docetaxel Treatment Initially Treated With Abiraterone Acetate vs Enzalutamide After Inverse Probability of Treatment Weighting **eFigure 7**. Hazard Ratios Over Time Among Patients With PSA Doubling Time of Less Than 3 Months Initially Treated With Abiraterone Acetate vs Enzalutamide After Inverse Probability of Treatment Weighting **eFigure 8**. Hazard Ratios Over Time Among Patients With Prior Docetaxel Treatment Initially Treated With Abiraterone Acetate vs Enzalutamide After Inverse Probability of Treatment Weighting **eFigure 9**. Hazard Ratios Over Time Among Hispanic Patients Initially Treated With Abiraterone Acetate vs Enzalutamide After Inverse Probability of Treatment Weighting **eFigure 10**. Hazard Ratios Over Time Among Non-Hispanic Black Patients Initially Treated With Abiraterone Acetate vs Enzalutamide After Inverse Probability of Treatment Weighting **eFigure 11**. Hazard Ratios Over Time Among Non-Hispanic White Patients Initially Treated With Abiraterone Acetate vs Enzalutamide After Inverse Probability of Treatment Weighting **eFigure 12**. Hazard Ratios Over Time Among Patients 75 Years or Older Initially Treated With Abiraterone Acetate vs Enzalutamide After Inverse Probability of Treatment Weighting **eFigure 13**. Hazard Ratios Over Time Among Patients Younger Than 75 Years Initially Treated With Abiraterone Acetate vs Enzalutamide After Inverse Probability of Treatment Weighting **eFigure 14.** Outcomes in Patients With Abiraterone Acetate and Enzalutamide Therapy With Greater Than 3 Months’ PSA Doubling Time **eFigure 15.** Outcomes in Patients With Abiraterone Acetate and Enzalutamide Therapy With No History of Prior Docetaxel Treatment **eFigure 16.** Outcomes in Patients With Abiraterone Acetate and Enzalutamide Therapy With Less Than 3 Months’ PSA Doubling Time **eFigure 17**. Outcomes in Patients With Abiraterone Acetate and Enzalutamide Therapy With History of Prior Docetaxel Treatment **eFigure 18**. Outcomes in Hispanic Patients Treated With Abiraterone Acetate and Enzalutamide Therapy **eFigure 19**. Outcomes in Non-Hispanic Black Patients Treated With Abiraterone Acetate and Enzalutamide Therapy **eFigure 20**. Outcomes in Non-Hispanic White Patients Treated With Abiraterone Acetate and Enzalutamide Therapy **eFigure 21**. Outcomes in Patients Aged 75 Years or Older Treated With Abiraterone Acetate and Enzalutamide Therapy **eFigure 22**. Outcomes in Patients Younger Than 75 Years Treated With Abiraterone Acetate and Enzalutamide Therapy

Supplement 2.
Data Sharing Statement
