# Supplementary material for: Abiraterone or Enzalutamide for Patients With Metastatic Castration-Resistant Prostate Cancer
Source: JAMA Netw Open. 2024 Aug 16;7(8):e2428444. doi: 10.1001/jamanetworkopen.2024.28444 (PMC11329885; doi:10.1001/jamanetworkopen.2024.28444)
Supplement: Supplement 1. — eMethods. eTable. RMST and Difference in RMST Between Enzalutamide Initiators and Abiraterone Acetate Initiators at 12, 24, 36, and 48 Months After Index Date for OS, PCS, TTS, and TTR eFigure 1. Standardized Mean Differences of Variables Before Inverse Probability of Treatment Weighting and After Weighting eFigure 2. Distribution of the Propensity Score for Patients Initially Treated With Abiraterone and Enzalutamide in the Unweighted Dataset eFigure 3. Inverse Probability of Treatment Weights for Patients Initially Treated With Abiraterone and Enzalutamide in the Unweighted Dataset eFigure 4. Hazard Ratios Over Time in the Full Cohort for Patients Initially Treated With Abiraterone Acetate vs Enzalutamide After Inverse Probability of Treatment Weighting eFigure 5. Hazard Ratios Over Time Among Patients With PSA Doubling Time of 3 Months or More Initially Treated With Abiraterone Acetate vs Enzalutamide After Inverse Probability of Treatment Weighting eFigure 6. Hazard Ratios Over Time Among Patients With No Prior Docetaxel Treatment Initially Treated With Abiraterone Acetate vs Enzalutamide After Inverse Probability of Treatment Weighting eFigure 7. Hazard Ratios Over Time Among Patients With PSA Doubling Time of Less Than 3 Months Initially Treated With Abiraterone Acetate vs Enzalutamide After Inverse Probability of Treatment Weighting eFigure 8. Hazard Ratios Over Time Among Patients With Prior Docetaxel Treatment Initially Treated With Abiraterone Acetate vs Enzalutamide After Inverse Probability of Treatment Weighting eFigure 9. Hazard Ratios Over Time Among Hispanic Patients Initially Treated With Abiraterone Acetate vs Enzalutamide After Inverse Probability of Treatment Weighting eFigure 10. Hazard Ratios Over Time Among Non-Hispanic Black Patients Initially Treated With Abiraterone Acetate vs Enzalutamide After Inverse Probability of Treatment Weighting eFigure 11. Hazard Ratios Over Time Among Non-Hispanic White Patients Initially Treated With Abiraterone [file jamanetwopen-e2428444-s001.pdf]

Supplementary Online Content

La J, Wang L, Corrigan JK, et al. Abiraterone or enzalutamide for patients with metastatic castration-resistant prostate cancer. *JAMA Netw Open*. 2024;7(8):e2428444. doi:10.1001/jamanetworkopen.2024.28444

eMethods
eTable. RMST and Difference in RMST Between Enzalutamide Initiators and Abiraterone Acetate Initiators at 12, 24, 36, and 48 Months After Index Date for OS, PCS, TTS, and TTR
eFigure 1. Standardized Mean Differences of Variables Before Inverse Probability of Treatment Weighting and After Weighting
eFigure 2. Distribution of the Propensity Score for Patients Initially Treated With Abiraterone and Enzalutamide in the Unweighted Dataset
eFigure 3. Inverse Probability of Treatment Weights for Patients Initially Treated With Abiraterone and Enzalutamide in the Unweighted Dataset
eFigure 4. Hazard Ratios Over Time in the Full Cohort for Patients Initially Treated With Abiraterone Acetate vs Enzalutamide After Inverse Probability of Treatment Weighting
eFigure 5. Hazard Ratios Over Time Among Patients With PSA Doubling Time of 3 Months or More Initially Treated With Abiraterone Acetate vs Enzalutamide After Inverse Probability of Treatment Weighting
eFigure 6. Hazard Ratios Over Time Among Patients With No Prior Docetaxel Treatment Initially Treated With Abiraterone Acetate vs Enzalutamide After Inverse Probability of Treatment Weighting
eFigure 7. Hazard Ratios Over Time Among Patients With PSA Doubling Time of Less Than 3 Months Initially Treated With Abiraterone Acetate vs Enzalutamide After Inverse Probability of Treatment Weighting
eFigure 8. Hazard Ratios Over Time Among Patients With Prior Docetaxel Treatment Initially Treated With Abiraterone Acetate vs Enzalutamide After Inverse Probability of Treatment Weighting
eFigure 9. Hazard Ratios Over Time Among Hispanic Patients Initially Treated With Abiraterone Acetate vs Enzalutamide After Inverse Probability of Treatment Weighting
eFigure 10. Hazard Ratios Over Time Among Non-Hispanic Black Patients Initially Treated With Abiraterone Acetate vs Enzalutamide After Inverse Probability of Treatment Weighting
eFigure 11. Hazard Ratios Over Time Among Non-Hispanic White Patients Initially Treated With Abiraterone Acetate vs Enzalutamide After Inverse Probability of Treatment Weighting
eFigure 12. Hazard Ratios Over Time Among Patients 75 Years or Older Initially Treated With Abiraterone Acetate vs Enzalutamide After Inverse Probability of Treatment Weighting

© 2024 La J et al. *JAMA Network Open*.

eFigure 13. Hazard Ratios Over Time Among Patients Younger Than 75 Years Initially Treated With Abiraterone Acetate vs Enzalutamide After Inverse Probability of Treatment Weighting
eFigure 14. Outcomes in Patients With Abiraterone Acetate and Enzalutamide Therapy With Greater Than 3 Months' PSA Doubling Time
eFigure 15. Outcomes in Patients With Abiraterone Acetate and Enzalutamide Therapy With No History of Prior Docetaxel Treatment
eFigure 16. Outcomes in Patients With Abiraterone Acetate and Enzalutamide Therapy With Less Than 3 Months' PSA Doubling Time
eFigure 17. Outcomes in Patients With Abiraterone Acetate and Enzalutamide Therapy With History of Prior Docetaxel Treatment
eFigure 18. Outcomes in Hispanic Patients Treated With Abiraterone Acetate and Enzalutamide Therapy
eFigure 19. Outcomes in Non-Hispanic Black Patients Treated With Abiraterone Acetate and Enzalutamide Therapy
eFigure 20. Outcomes in Non-Hispanic White Patients Treated With Abiraterone Acetate and Enzalutamide Therapy
eFigure 21. Outcomes in Patients Aged 75 Years or Older Treated With Abiraterone Acetate and Enzalutamide Therapy
eFigure 22. Outcomes in Patients Younger Than 75 Years Treated With Abiraterone Acetate and Enzalutamide Therapy

This supplementary material has been provided by the authors to give readers additional information about their work.

© 2024 La J et al. *JAMA Network Open*.

eMethods

Outcome Variable Details

The index date and censoring criteria are described in the main text.

Overall survival: Death from any cause, as recorded in the VA CDW.

Prostate-cancer-specific survival: Death from prostate cancer (ICD-10 Code C61*) as recorded on the death certificate from the National Death Index and linked to VA patient identifiers using the VA Mortality Data Repository.

Treatment switching or death: The first occurrence of a treatment switch or any cause of death. Prostate cancer treatments used to define treatment switching or death are as follows: abiraterone, enzalutamide, nilutamide, flutamide, bicalutamide, ketoconazole, docetaxel, cabazitaxel, mitoxantrone, carboplatin, olaparib, talazoparib, rucaparib, radium 223, sipuleucel-T, Lu 177, pembrolizumab.

PSA response: The first occurrence of PSA levels ≤50% of baseline PSA value.

Baseline Variable Details

Age: Age at index date was defined based on birth date recorded in the CDW.

Sex: Sex is as recorded in the CDW.

Race/Ethnicity: Race/ethnicity was extracted from the CDW. Race and ethnicity data in the CDW is self-reported data as recorded in the electronic health record using pre-defined categories for race and ethnicity. Categories for race were American Indian or Alaska Native, Asian, Black or African American, Declined to Answer, Native Hawaiian or Other Pacific Islander, Unknown by Patient, White, White not of Hisp Origin. Categories for ethnicity were Declined to Answer, Hispanic or Latino, Not Hispanic or Latino, Unknown by Patient. Data was recoded as Hispanic, Non-Hispanic Black, Non-Hispanic White, or Other/Unknown. Thus, the

© 2024 La J et al. *JAMA Network Open*.

Other/Unknown category includes American Indian or Alaska Native, Asian, Native Hawaiian or Other Pacific Islander, Declined to Answer, and Unknown by Patient.

Comorbidities: Individual comorbidities were measured using definitions from the Centers for Medicare & Medicaid Services (CMS) Chronic Conditions Warehouse[23] based on both diagnosis and procedure codes recorded in CDW in the three years prior to the index date.

Frailty: Frailty was measured using the VA Frailty Index,[22] an electronic frailty index based on the deficit-accumulation approach. Thirty-one aging-related health deficits are measured from electronic health record data and administrative claims, using diagnostic and procedural codes in the three years prior to the index date, and a score is calculated for each patient as the proportion of possible health deficits that are present in an individual.

Prior treatments: Prior treatments (docetaxel, radiation therapy, prostatectomy, bisphosphonate) were defined as occurrence of any of these treatments prior to the index date. Prior treatment was not limited to a lookback window.

Baseline PSA levels: Data captured from the VA CDW using the closest PSA value within a 365-day lookback window before the index date.

Pre-therapy hemoglobin levels: Data captured from the VA CDW using the closest hemoglobin value within a 90-day lookback window before the index date.

Pre-therapy LDH levels: Data captured from the VA CDW using the closest LDH value within a 90-day lookback window before the index date.

PSA doubling time: ln(2) / PSA slope of the last three PSA values within 365 days prior to the index date. The PSA slope (i.e., the slope of the log-transformed PSA over time) was estimated using a linear model. If two tests were detected on the same day, the mean PSA value for that day was used. There was no restriction on the number of days apart for the PSA values, provided that they fall within the 365-day lookback window.

© 2024 La J et al. *JAMA Network Open*.

Prior orchiectomy: A record of at least one of the following codes prior to the index date. CPT Codes: 54520, 54522, 54530, 54535; ICD-9 procedure codes: 62.3, 62.4, 62.41, 62.42; and ICD-10 procedure codes: 0VT90ZZ, 0VT94ZZ, 0VTB0ZZ, 0VTB4ZZ, 0VTC0ZZ, 0VTC4ZZ.

Prior androgen-deprivation treatment: A record of at least one of the following treatments: goserelin, leuprolide, triptorelin, histrelin, bicalutamide, flutamide, and/or nilutamide.

Annotation Protocol for Metastatic Status Chart Review

The natural language processing algorithm[19] identifies the note used to determine metastatic disease. These notes were extracted for the 7,996 patients who were determined by the algorithm to have metastatic disease before or up to 30 days after abiraterone acetate or enzalutamide initiation. Notes were randomly assigned to two clinicians for chart review, and a final determination as to metastatic status was made by the reviewer.

© 2024 La J et al. *JAMA Network Open*.

eTable 1. Restricted mean survival time (RMST) and the difference in RMST between enzalutamide initiators and abiraterone acetate initiators at 12, 24, 36, and 48 months after the index date for overall survival (OS), prostate cancer-specific survival (PCS), time to treatment switching or death (TTS), and time to prostate-specific antigen response (TTR).

Months	Outcome	RMST among Abiraterone Acetate Initiators	RMST Among Enzalutamide Initiatiors	RMST Difference (95% CI)
12	OS	10.33 (10.22-10.43)	10.59 (10.47-10.72)	0.27 (0.11-0.43)
24	OS	16.97 (16.70-17.23)	17.50 (17.16-17.83)	0.53 (0.10-0.96)
36	OS	20.98 (20.56-21.39)	21.76 (21.22-22.29)	0.78 (0.10-1.46)
48	OS	23.38 (22.85-23.92)	24.29 (23.58-24.99)	0.90 (0.02-1.79)
12	PCS	10.66 (10.55-10.77)	10.91 (10.78-11.04)	0.25 (0.09-0.42)
24	PCS	18.43 (18.14-18.72)	18.91 (18.54-19.28)	0.48 (0.01-0.95)
36	PCS	24.33 (23.84-24.81)	24.95 (24.32-25.59)	0.62 (-0.18-1.42)
48	PCS	29.08 (28.39-29.76)	29.78 (28.88-30.69)	0.71 (-0.43-1.84)
12	TTS	8.71 (8.57-8.86)	9.31 (9.14-9.49)	0.60 (0.37-0.82)
24	TTS	13.06 (12.74-13.38)	14.27 (13.86-14.69)	1.21 (0.69-1.74)
36	TTS	15.29 (14.82-15.76)	17.01 (16.37-17.65)	1.72 (0.93-2.51)
48	TTS	16.67 (16.06-17.28)	18.62 (17.79-19.46)	1.95 (0.92-2.99)
12	TTR	6.32 (6.14-6.50)	4.98 (4.76-5.21)	-1.34 (-1.63--1.05)
24	TTR	10.08 (9.65-10.51)	7.98 (7.46-8.50)	-2.10 (-2.78--1.42)
36	TTR	13.50 (12.71-14.28)	10.56 (9.58-11.55)	-2.93 (-4.19--1.67)
48	TTR	16.20 (15.07-17.34)	12.64 (11.22-14.05)	-3.57 (-5.38--1.76)

© 2024 La J et al. *JAMA Network Open*.

eFigure 1. Standardized mean differences (SMD) of variables before inverse probability of treatment weighting (blue line) and after (red line) weighting. The grey line at 0.1 shows the conventional threshold for SMD of well-balanced variables.

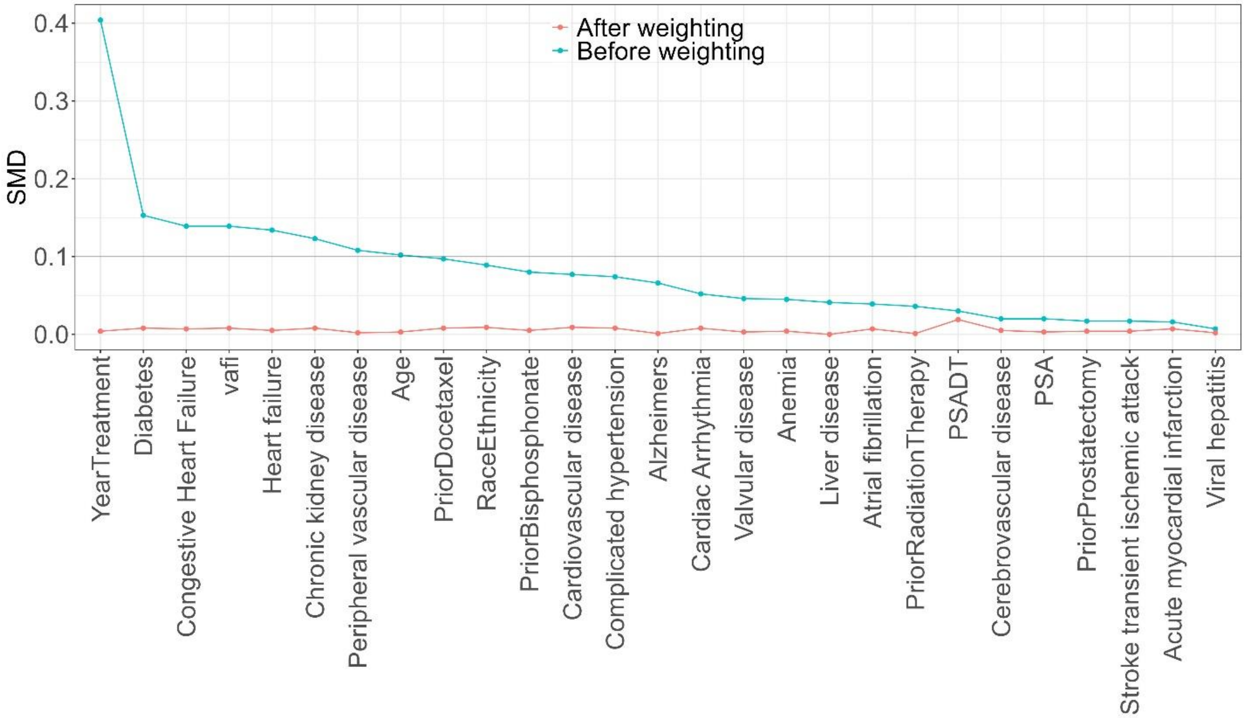

© 2024 La J et al. *JAMA Network Open*.

eFigure 2. Distribution of the propensity score for patients initially treated with abiraterone and enzalutamide in the unweighted dataset. For each bin, the height of the bar shows the number of patients with a propensity score falling in that bin.

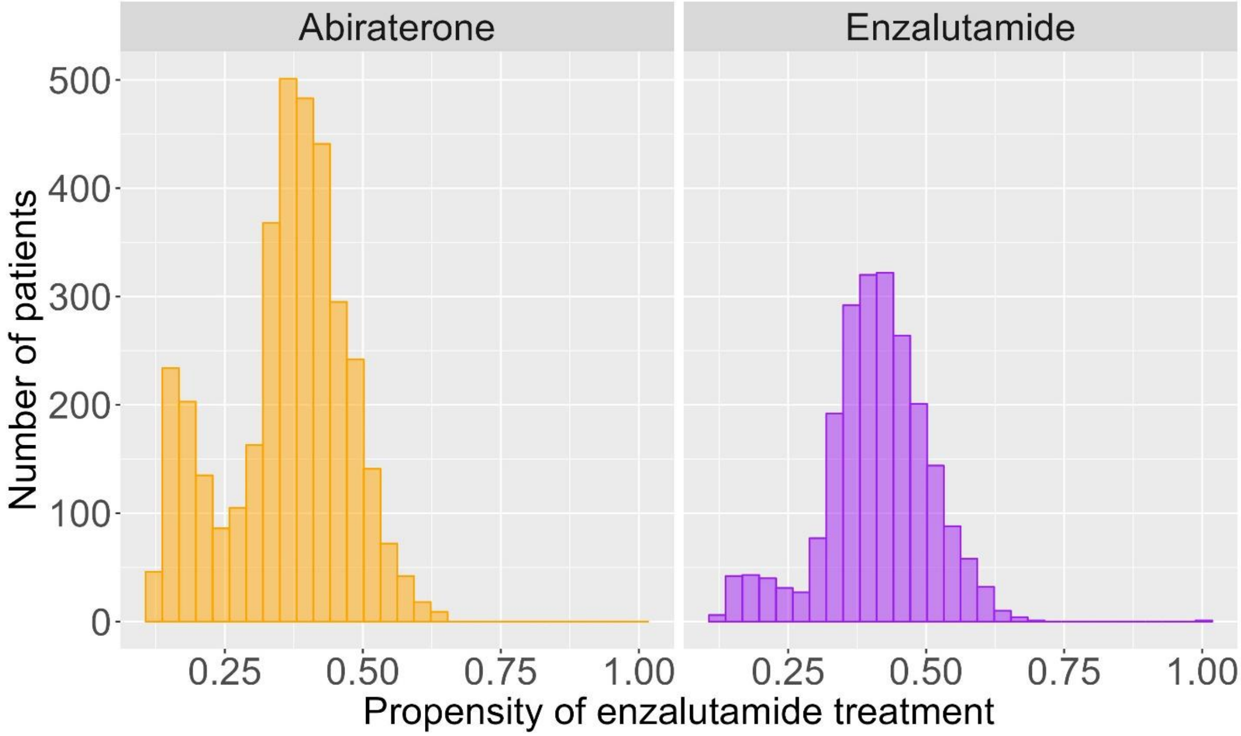

© 2024 La J et al. *JAMA Network Open*.

eFigure 3. Inverse probability of treatment weights for patients initially treated with abiraterone and enzalutamide in the unweighted dataset. For each bin, the height of the bar shows the number of patients with a stabilized weight score falling in that bin.

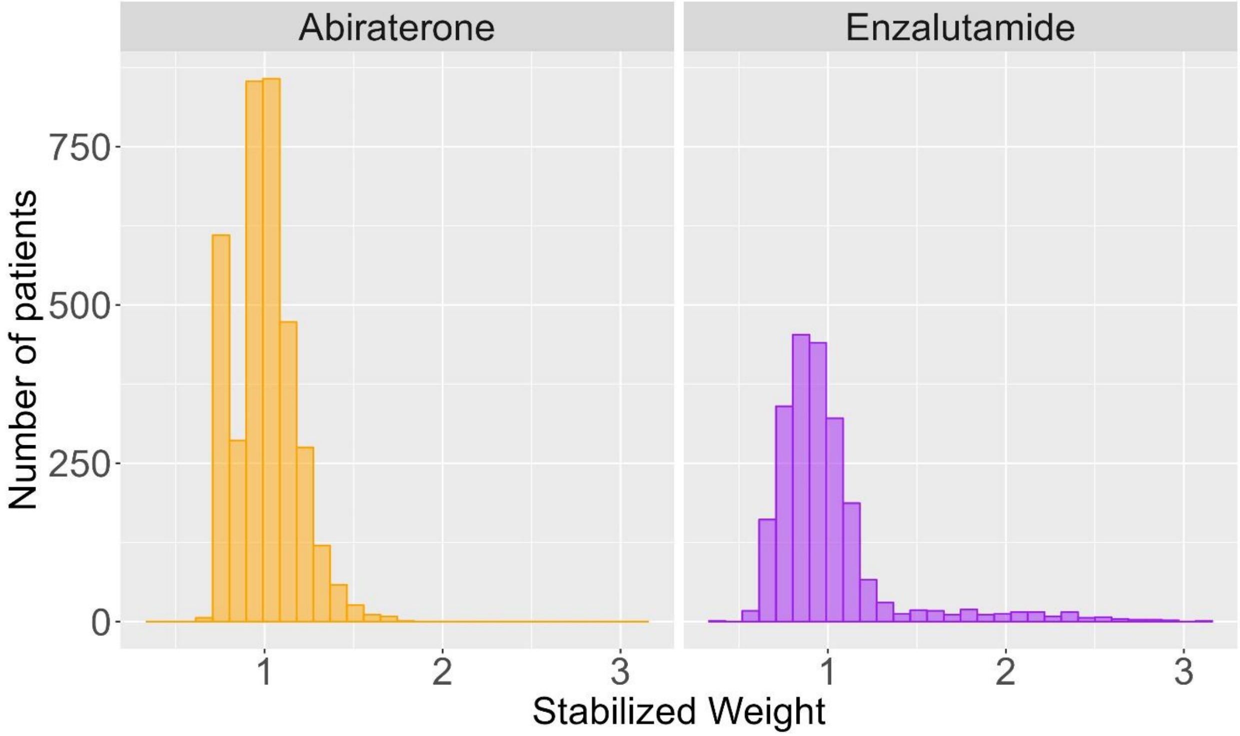

© 2024 La J et al. *JAMA Network Open*.

eFigure 4. Hazard ratios over time in the full cohort for patients initially treated with abiraterone acetate (reference) versus enzalutamide after inverse probability of treatment weighting. P-values were obtained using Schoenfeld's global test.

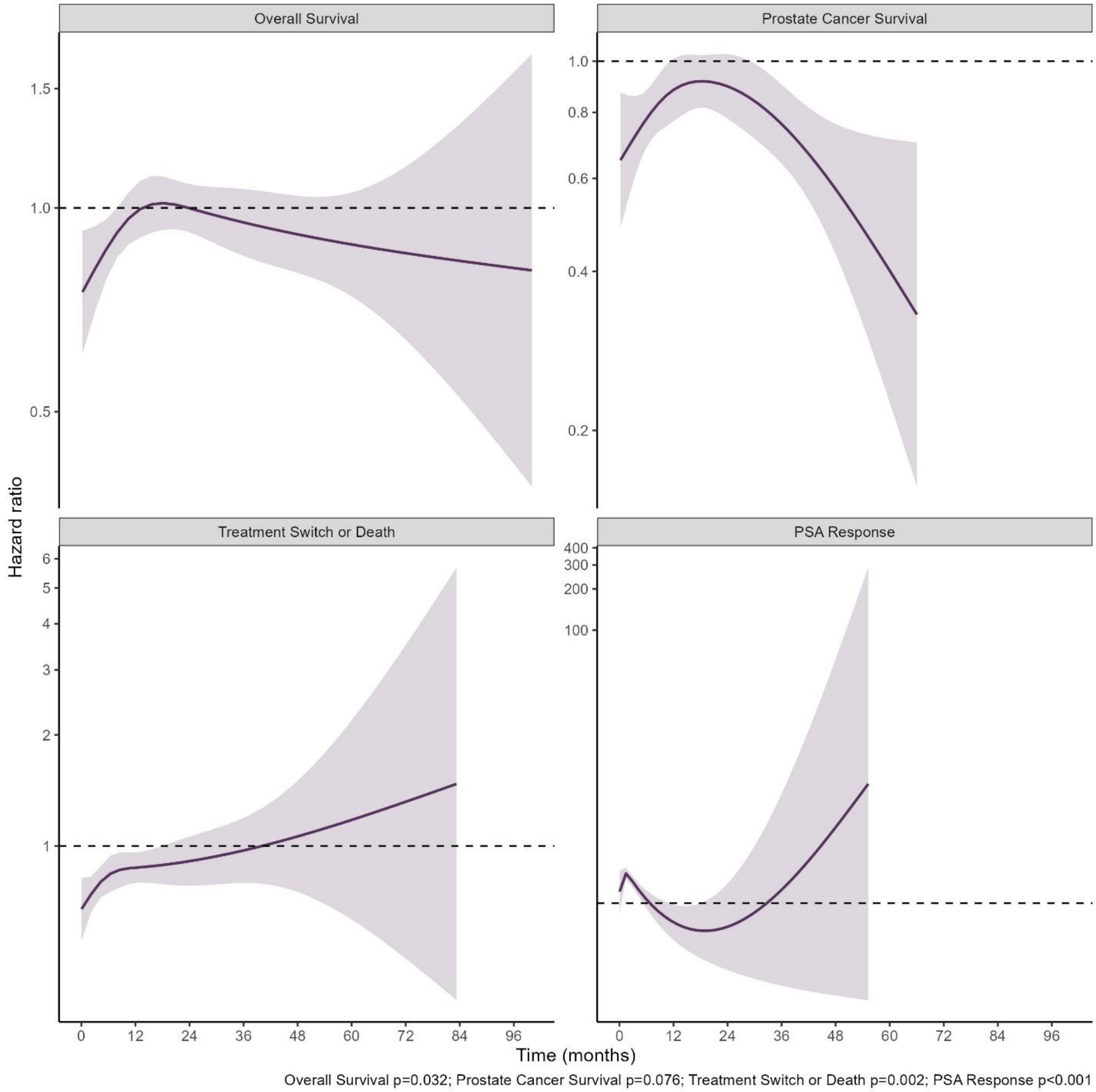

© 2024 La J et al. *JAMA Network Open*.

eFigure 5. Hazard ratios over time among patients with PSA doubling time ≥ 3 months who were initially treated with abiraterone acetate (reference) versus enzalutamide after inverse probability of treatment weighting. P-values were obtained using Schoenfeld's global test.

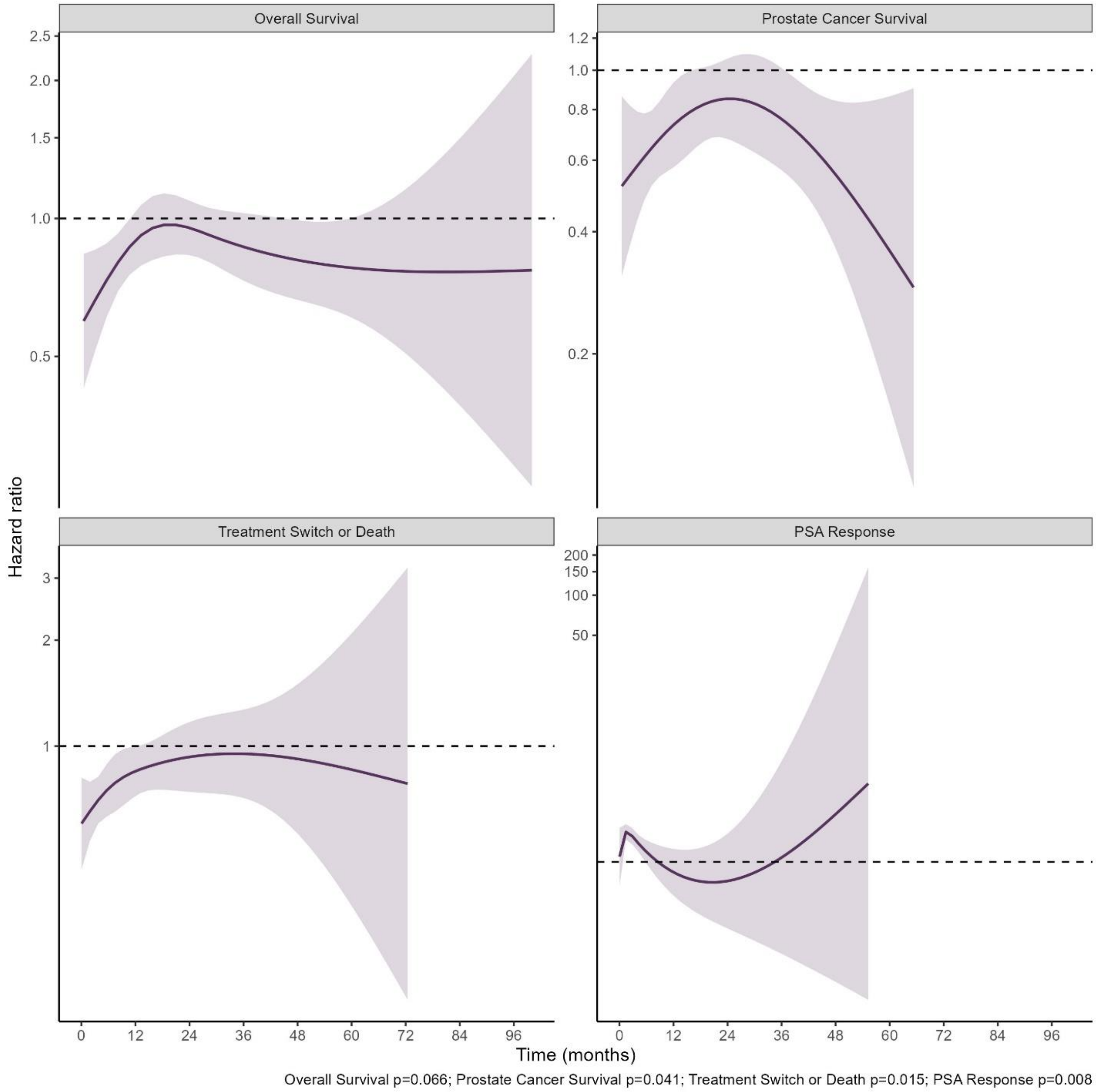

© 2024 La J et al. *JAMA Network Open*.

eFigure 6. Hazard ratios over time among patients with no prior docetaxel treatment who were initially treated with abiraterone acetate (reference) versus enzalutamide after inverse probability of treatment weighting. P-values were obtained using Schoenfeld's global test.

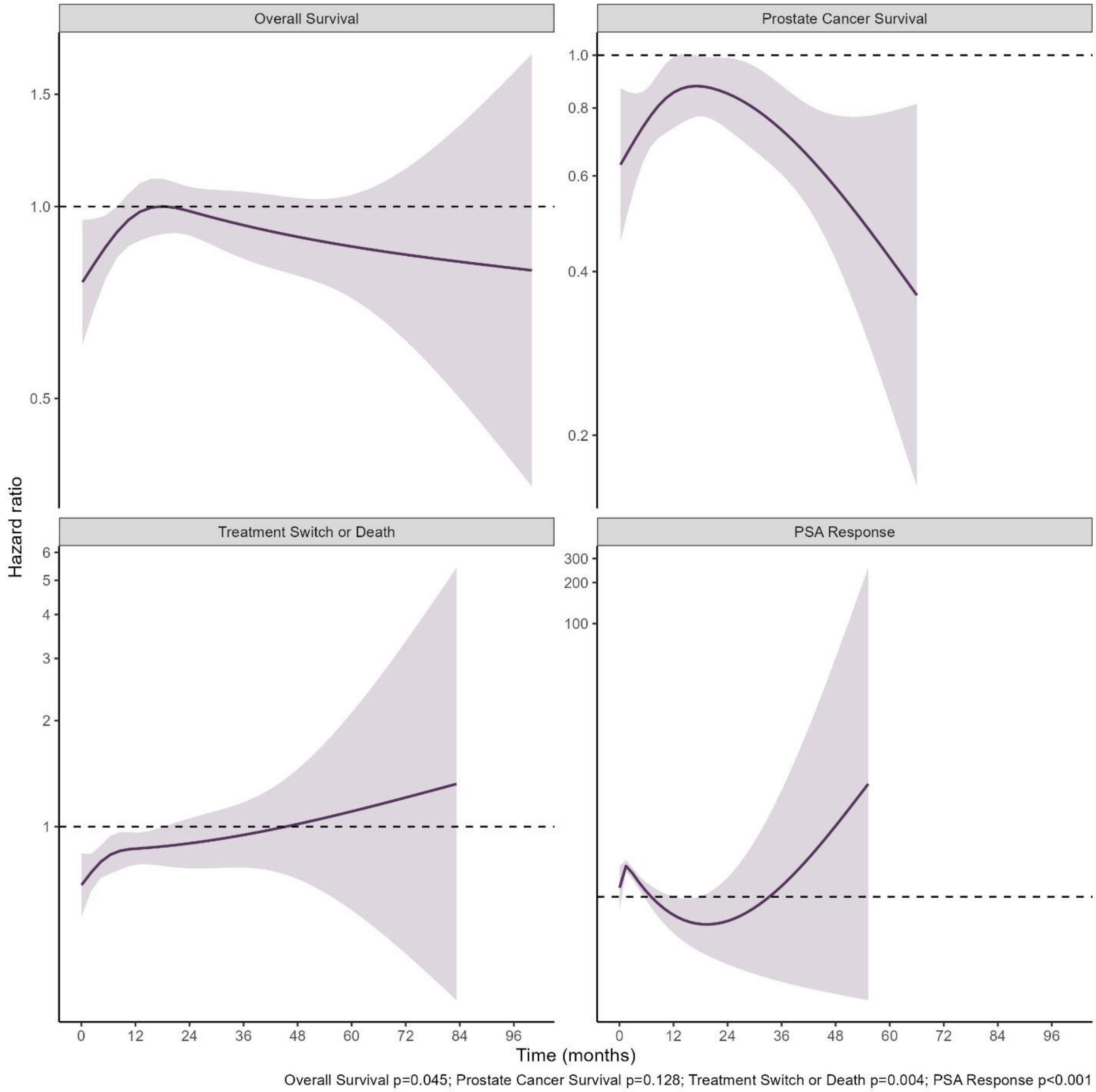

© 2024 La J et al. *JAMA Network Open*.

eFigure 7. Hazard ratios over time among patients with PSA doubling time < 3 months who were initially treated with abiraterone acetate (reference) versus enzalutamide after inverse probability of treatment weighting. P-values were obtained using Schoenfeld's global test.

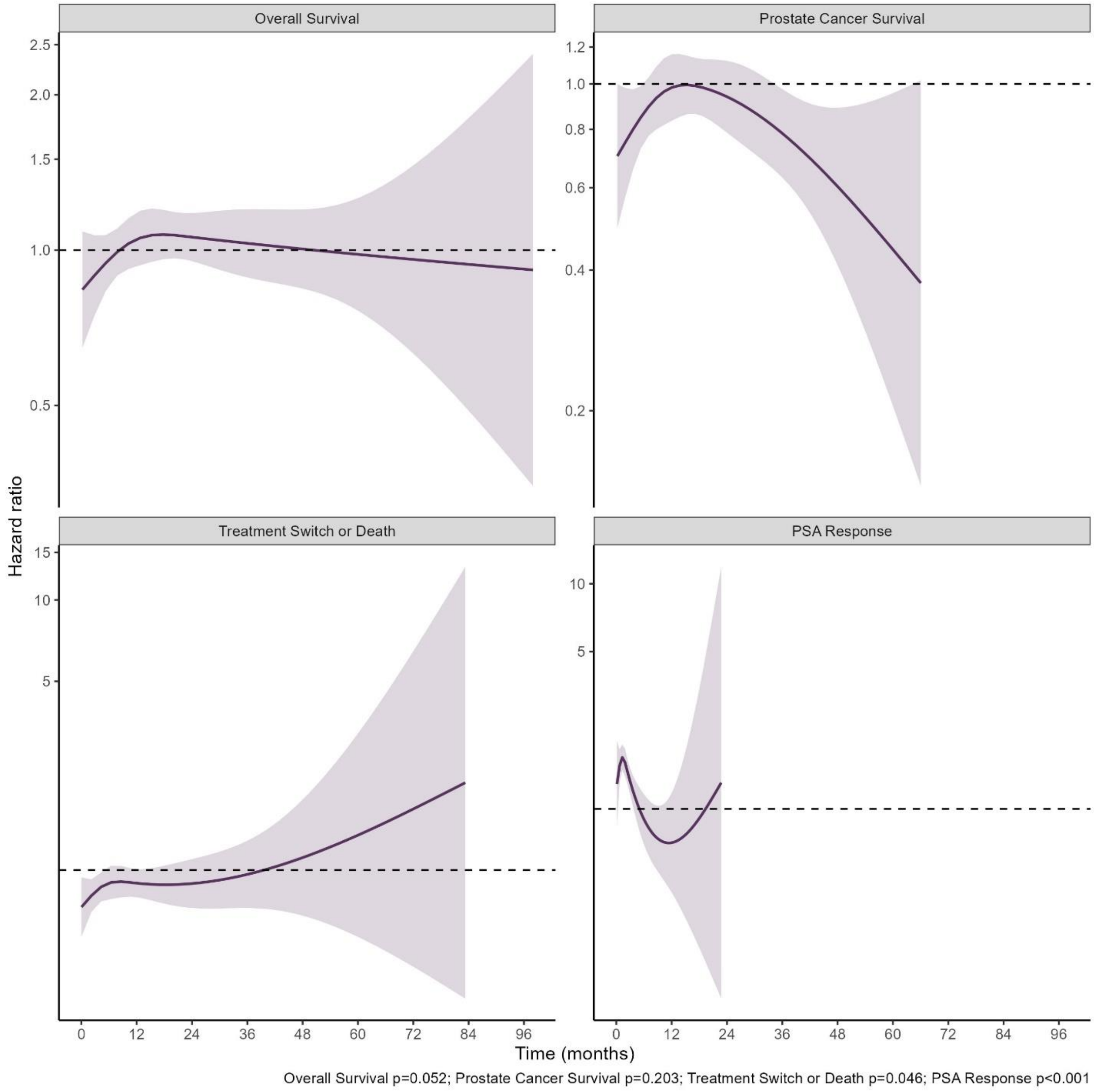

© 2024 La J et al. *JAMA Network Open*.

eFigure 8. Hazard ratios over time among patients with prior docetaxel treatment who were initially treated with abiraterone acetate (reference) versus enzalutamide after inverse probability of treatment weighting. P-values were obtained using Schoenfeld's global test.

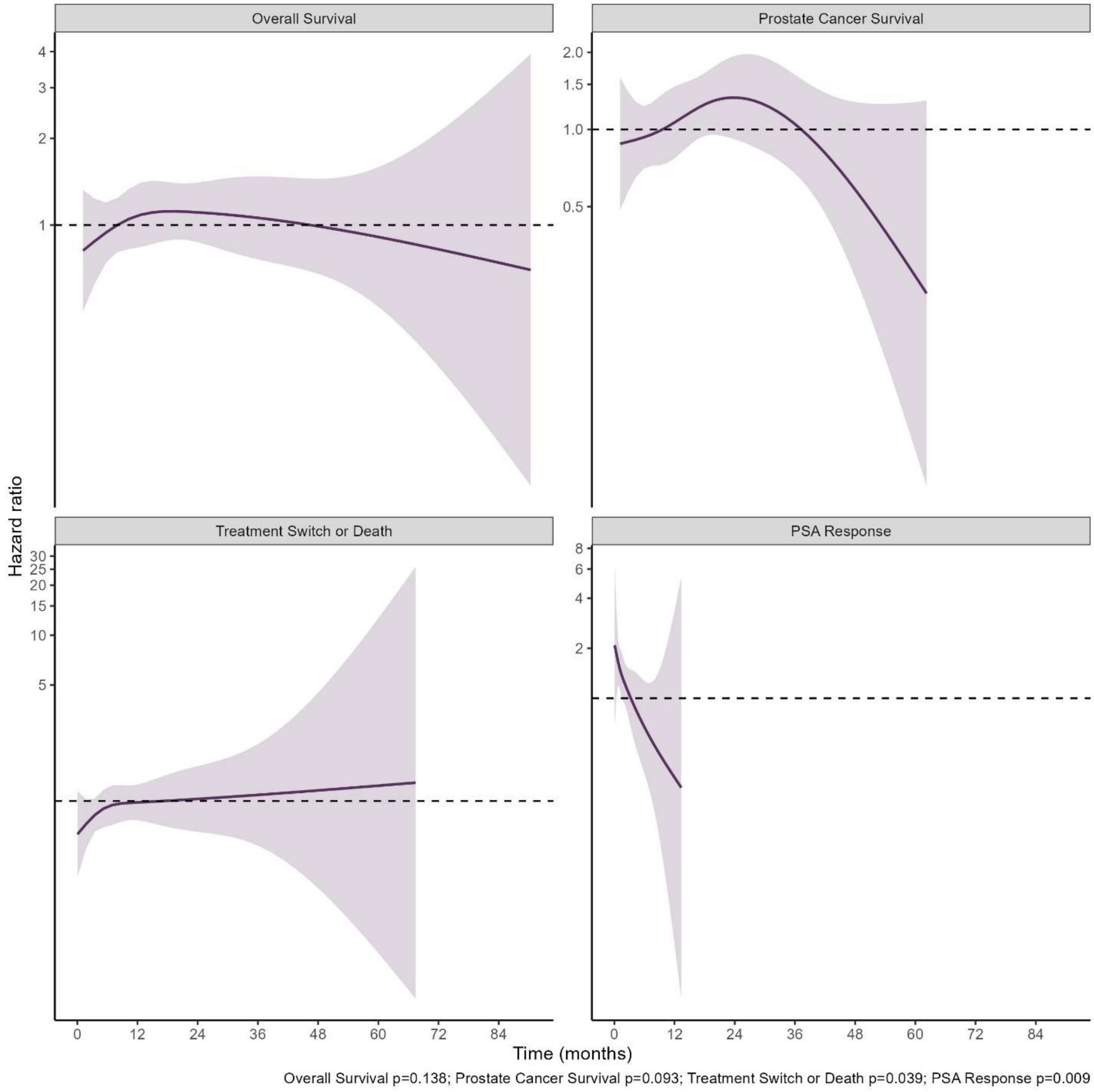

© 2024 La J et al. *JAMA Network Open*.

eFigure 9. Hazard ratios over time among Hispanic patients who were initially treated with abiraterone acetate (reference) versus enzalutamide after inverse probability of treatment weighting. P-values were obtained using Schoenfeld's global test.

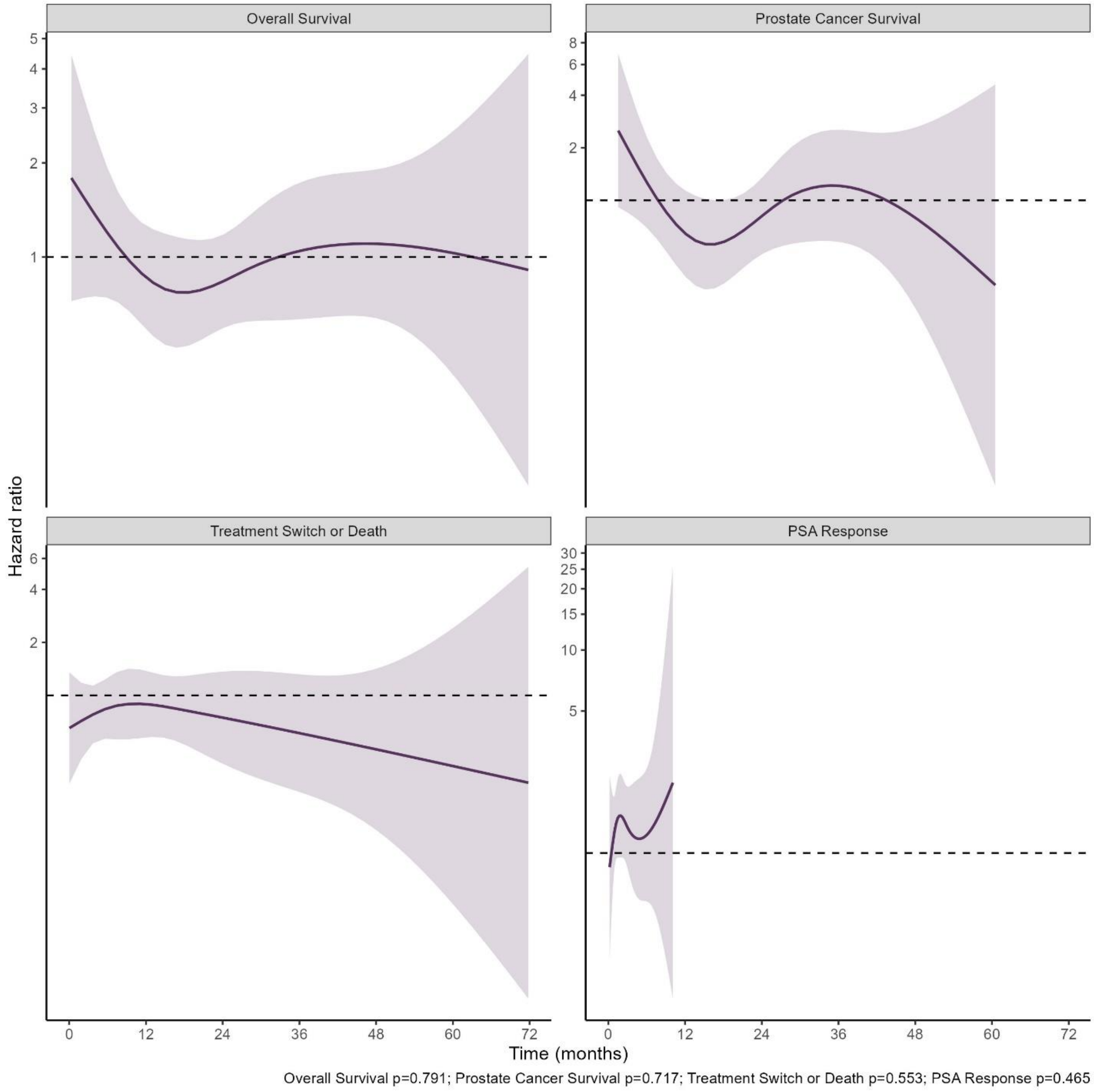

© 2024 La J et al. *JAMA Network Open*.

eFigure 10. Hazard ratios over time among Non-Hispanic Black patients who were initially treated with abiraterone acetate (reference) versus enzalutamide after inverse probability of treatment weighting. P-values were obtained using Schoenfeld's global test.

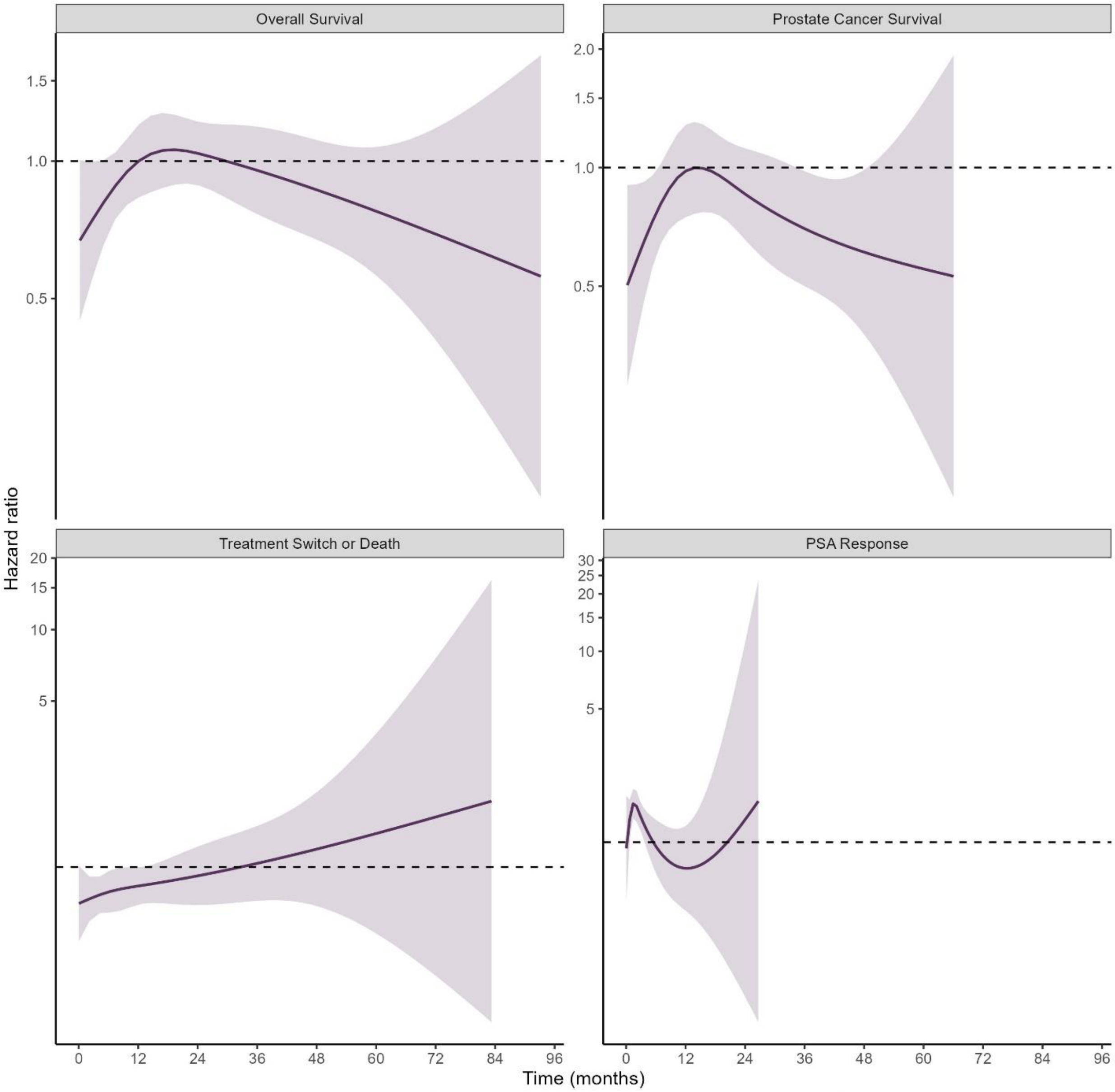

© 2024 La J et al. *JAMA Network Open*.

eFigure 11. Hazard ratios over time among Non-Hispanic White patients who were initially treated with abiraterone acetate (reference) versus enzalutamide after inverse probability of treatment weighting. P-values were obtained using Schoenfeld's global test.

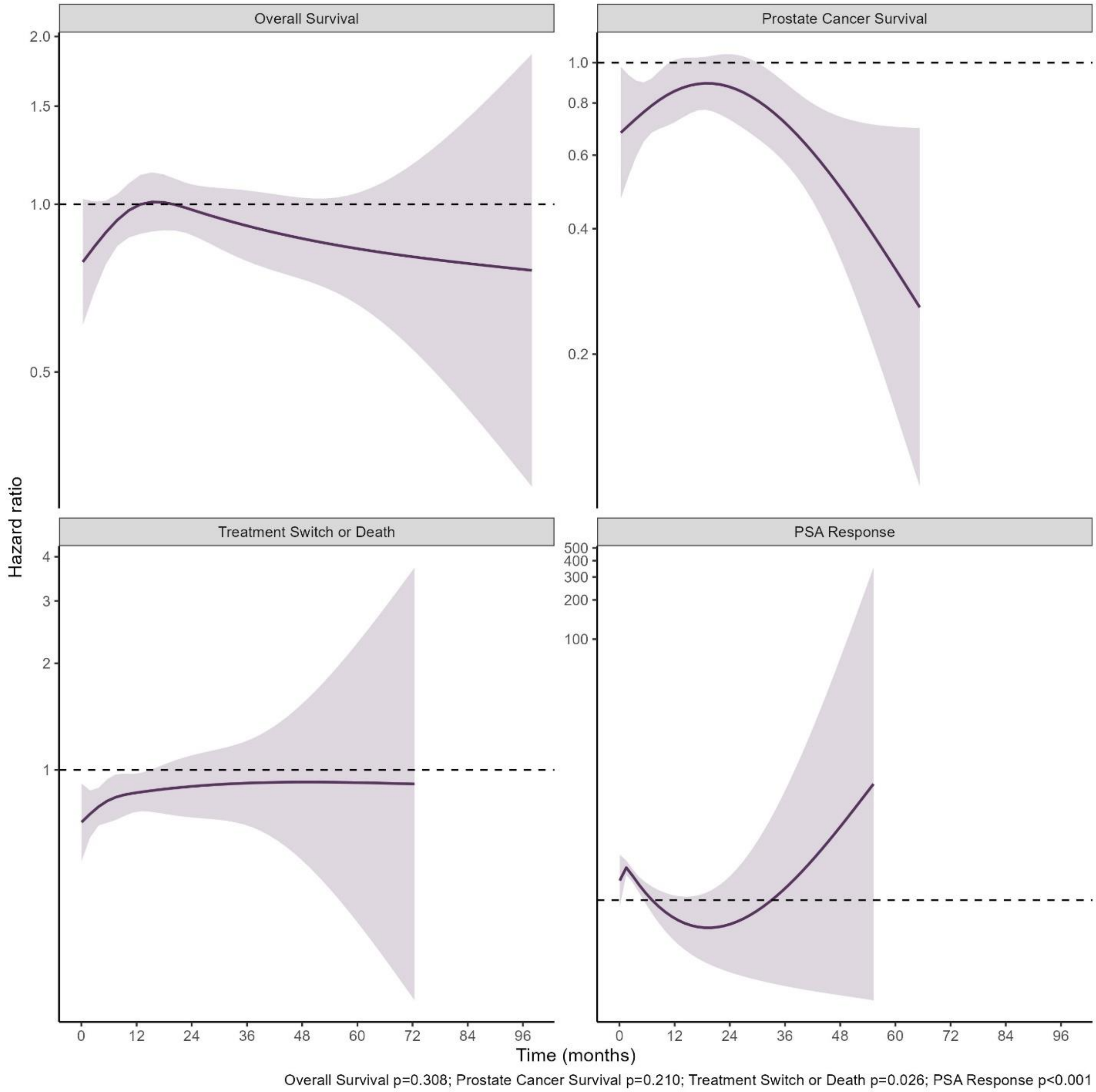

© 2024 La J et al. *JAMA Network Open*.

eFigure 12. Hazard ratios over time among patients 75 or older who were initially treated with abiraterone acetate (reference) versus enzalutamide after inverse probability of treatment weighting. P-values were obtained using Schoenfeld's global test.

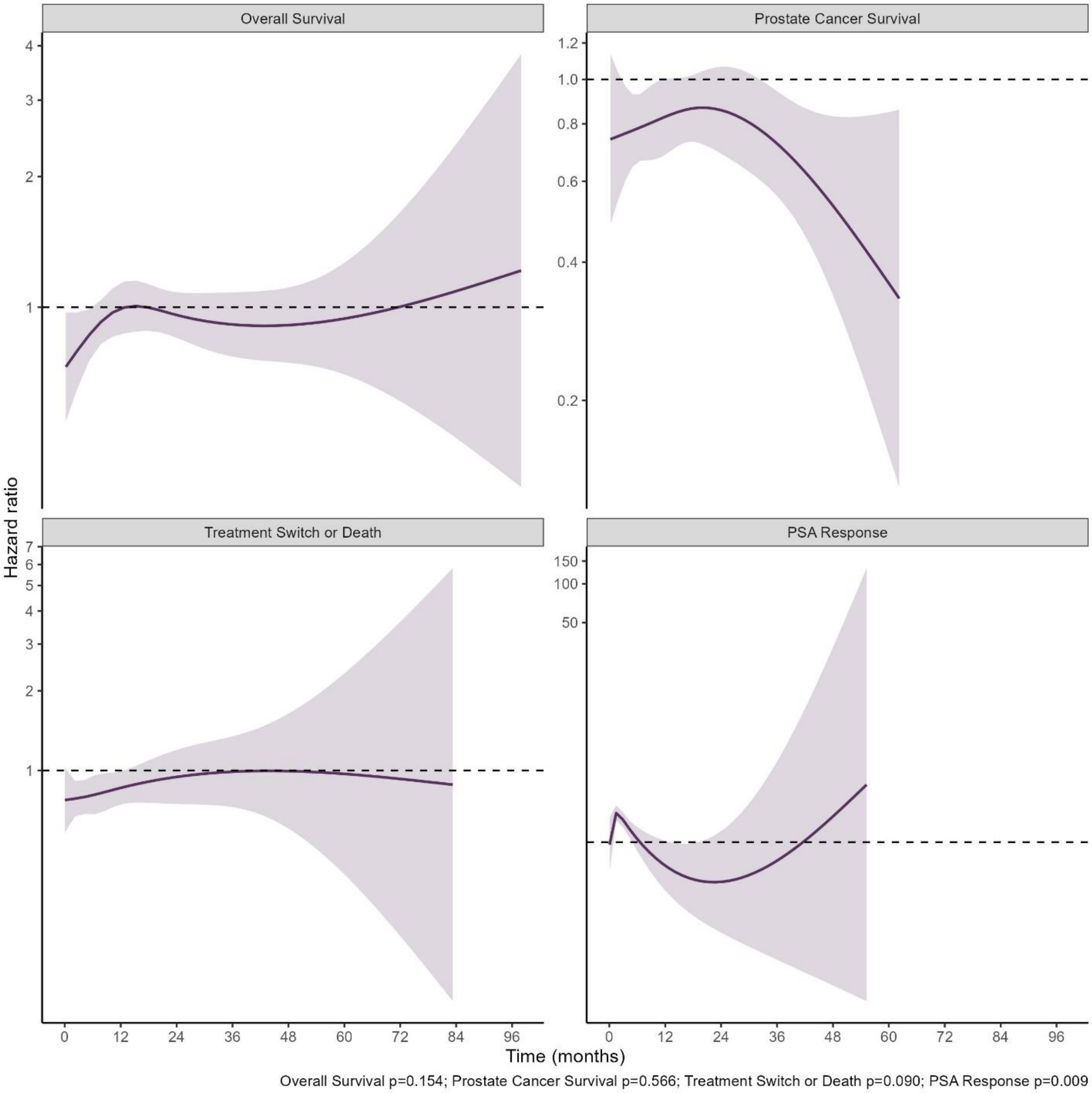

© 2024 La J et al. *JAMA Network Open*.

eFigure 13. Hazard ratios over time among patients younger than 75 who were initially treated with abiraterone acetate (reference) versus enzalutamide after inverse probability of treatment weighting. P-values were obtained using Schoenfeld's global test.

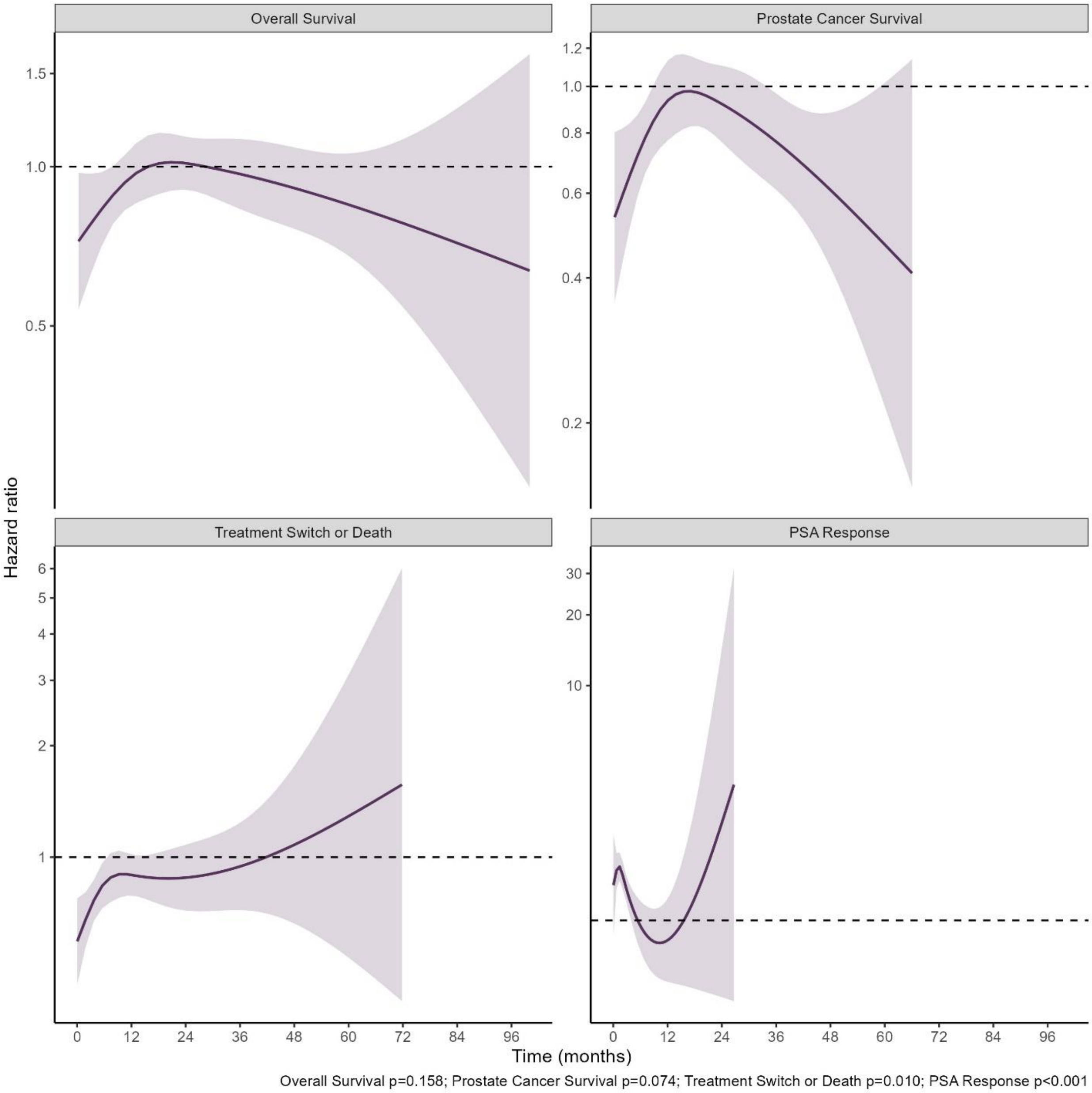

© 2024 La J et al. *JAMA Network Open*.

eFigure 14. Outcomes in patients with abiraterone acetate and enzalutamide therapy with ≥ 3 months PSA doubling time. In addition to Kaplan-Meier plots, the difference in restricted mean survival time (RMST) at each time point (three-month increments) is shown. The RMST at time *t* measures the mean survival censoring at *t*, and is equal to the area under the Kaplan-Meier plot up to *t*. The number of events and number at risk for abiraterone acetate (blue) or enzalutamide (orange) are tabulated under graphs at each timepoint. (A) Overall survival. (B) Prostate cancer survival. (C) Time to PSA response. (D) Time to treatment switch or death.

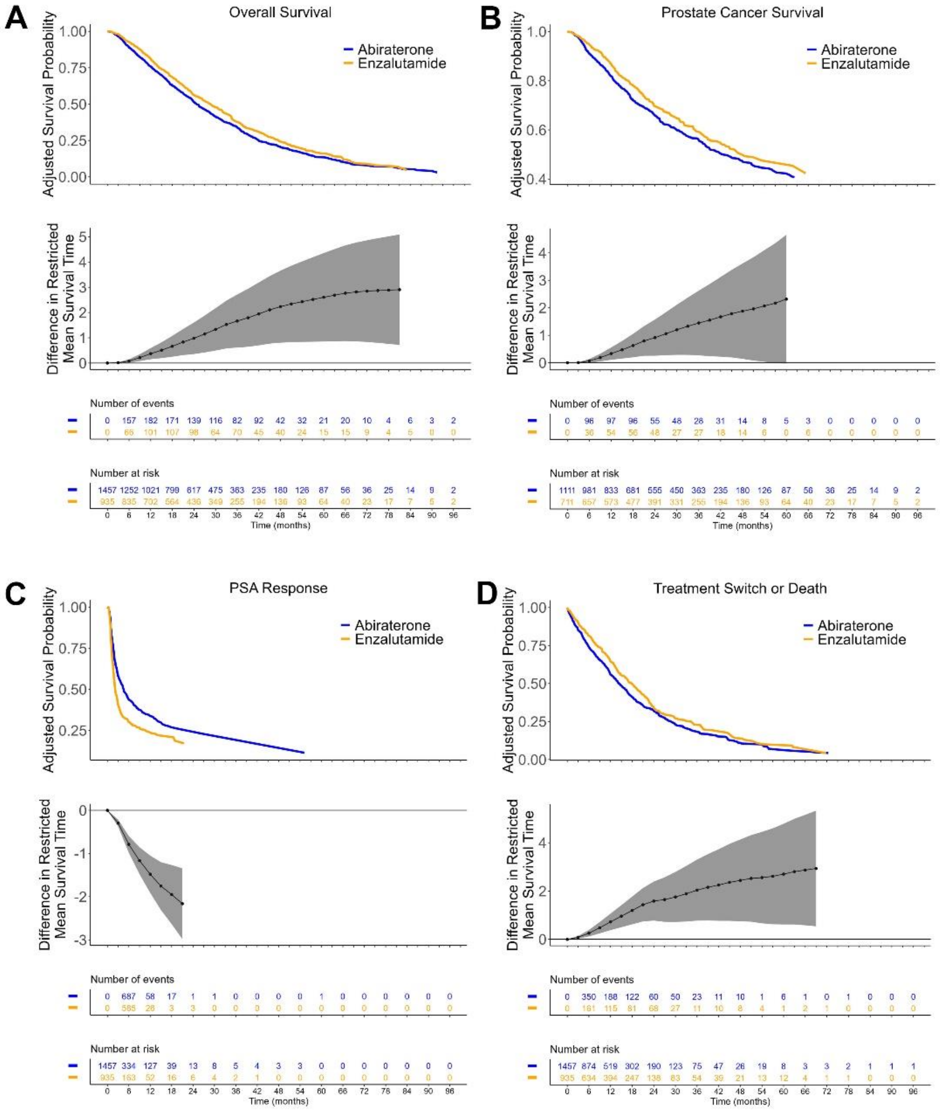

© 2024 La J et al. *JAMA Network Open*.

eFigure 15. Outcomes in patients with abiraterone acetate and enzalutamide therapy with no history of prior docetaxel treatment. In addition to Kaplan-Meier plots, the difference in restricted mean survival time (RMST) at each time point (three-month increments) is shown. The RMST at time *t* measures the mean survival censoring at *t*, and is equal to the area under the Kaplan-Meier plot up to *t*. The number of events and number at risk for abiraterone acetate (blue) or enzalutamide (orange) are tabulated under graphs at each timepoint. (A) Overall survival. (B) Prostate cancer survival. (C) Time to PSA response. (D) Time to treatment switch or death.

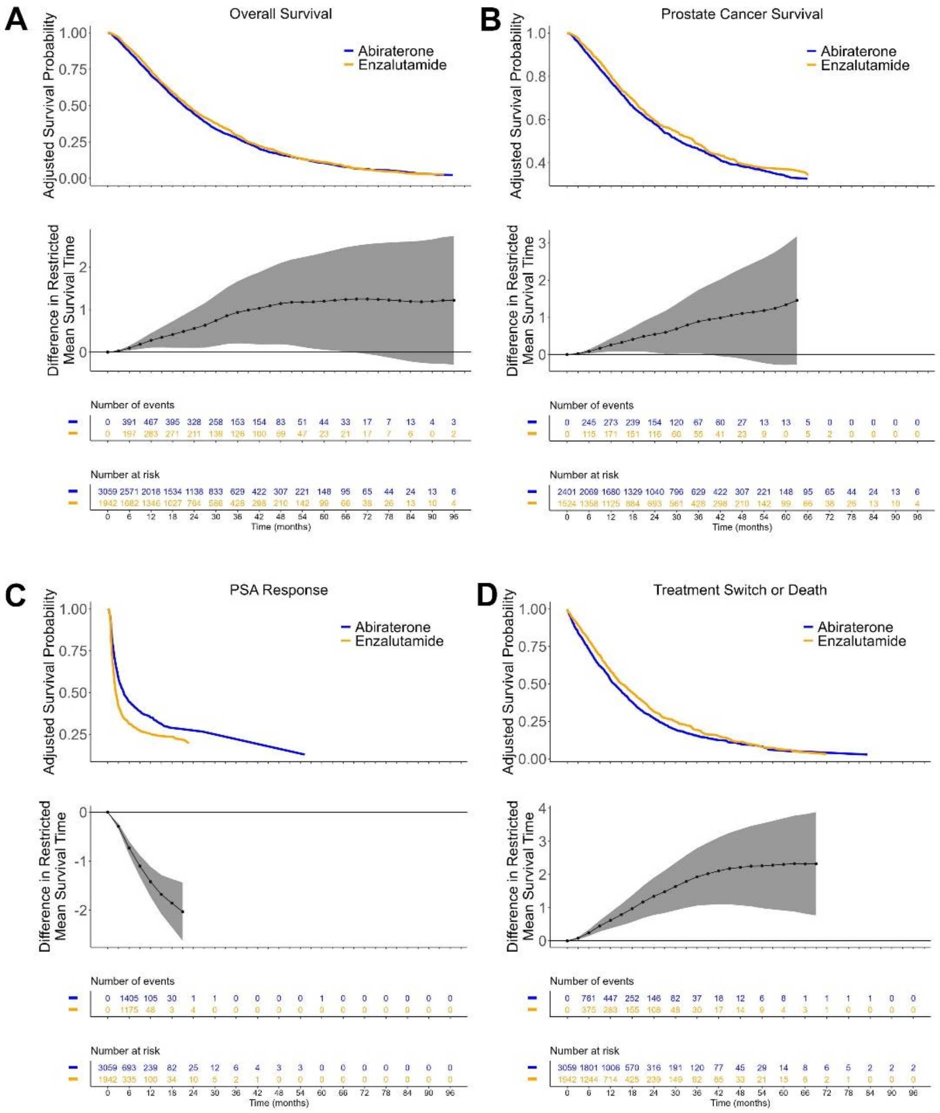

© 2024 La J et al. *JAMA Network Open*.

eFigure 16. Outcomes in patients with abiraterone acetate and enzalutamide therapy with < 3 months PSA doubling time. In addition to Kaplan-Meier plots, the difference in restricted mean survival time (RMST) at each time point (three-month increments) is shown. The RMST at time *t* measures the mean survival censoring at *t*, and is equal to the area under the Kaplan-Meier plot up to *t*. The number of events and number at risk for abiraterone acetate (blue) or enzalutamide (orange) are tabulated under graphs at each timepoint. (A) Overall survival. (B) Prostate cancer survival. (C) Time to PSA response. (D) Time to treatment switch or death.

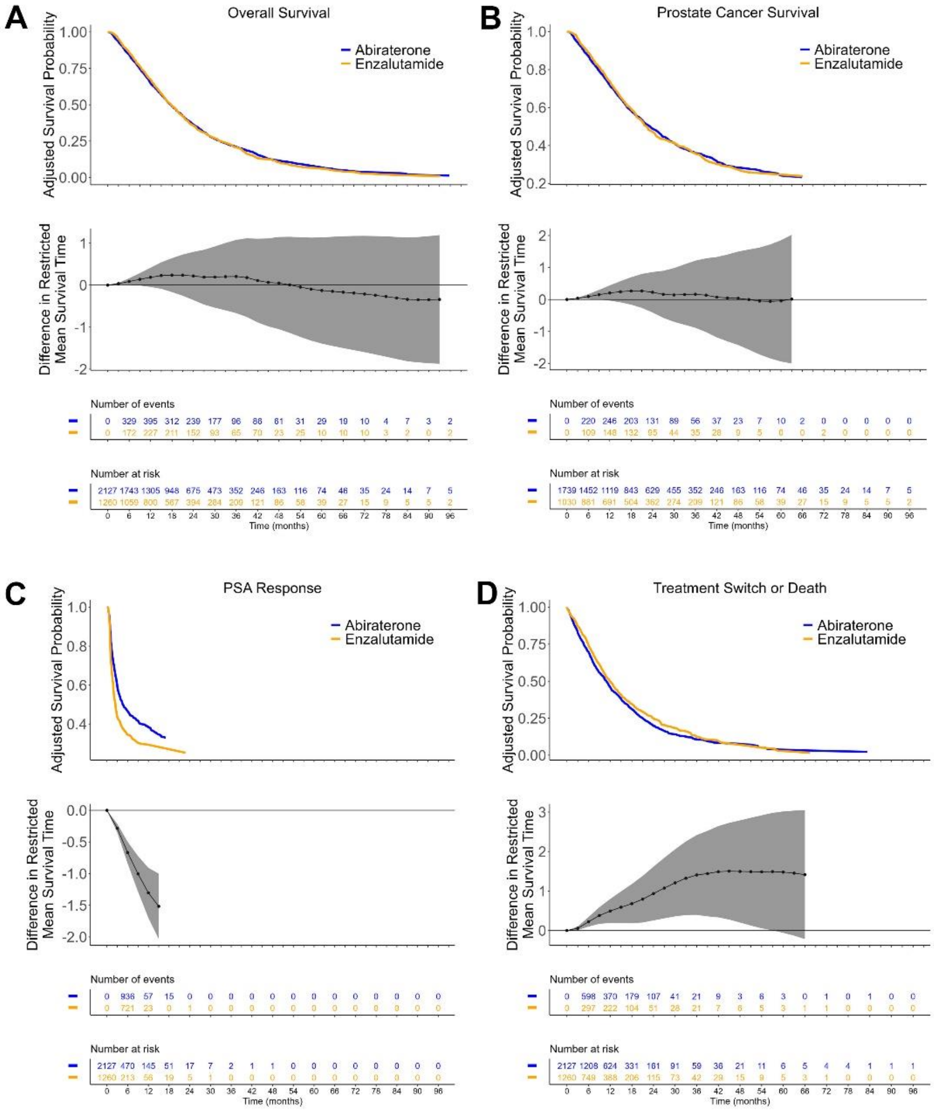

© 2024 La J et al. *JAMA Network Open*.

eFigure 17. Outcomes in patients with abiraterone acetate and enzalutamide therapy with history of prior docetaxel treatment. In addition to Kaplan-Meier plots, the difference in restricted mean survival time (RMST) at each time point (three-month increments) is shown. The RMST at time *t* measures the mean survival censoring at *t*, and is equal to the area under the Kaplan-Meier plot up to *t*. The number of events and number at risk for abiraterone acetate (blue) or enzalutamide (orange) are tabulated under graphs at each timepoint. (A) Overall survival. (B) Prostate cancer survival. (C) Time to PSA response. (D) Time to treatment switch or death.

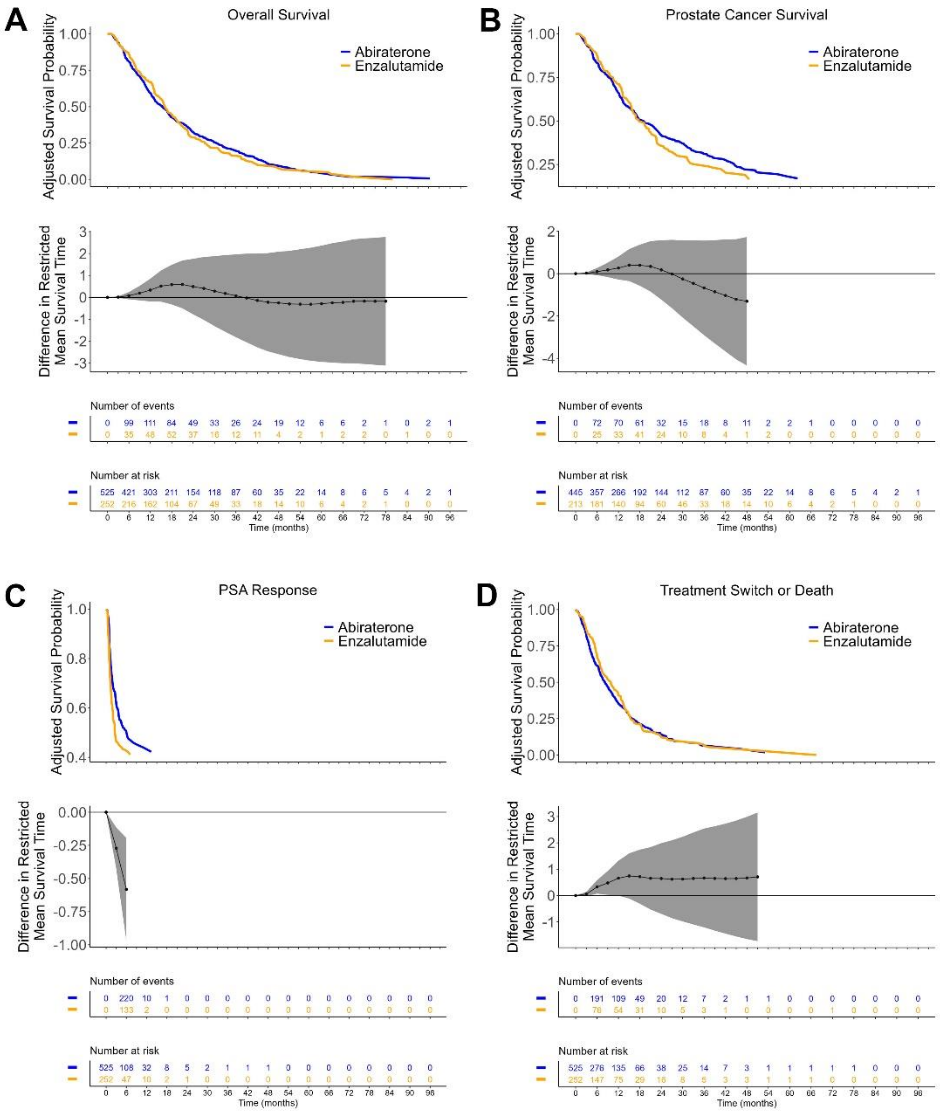

© 2024 La J et al. *JAMA Network Open*.

eFigure 18. Outcomes in Hispanic patients treated with abiraterone acetate and enzalutamide therapy. In addition to Kaplan-Meier plots, the difference in restricted mean survival time (RMST) at each time point (three-month increments) is shown. The RMST at time *t* measures the mean survival censoring at *t*, and is equal to the area under the Kaplan-Meier plot up to *t*. The number of events and number at risk for abiraterone acetate (blue) or enzalutamide (orange) are tabulated under graphs at each timepoint. (A) Overall survival. (B) Prostate cancer survival. (C) Time to PSA response. (D) Time to treatment switch or death.

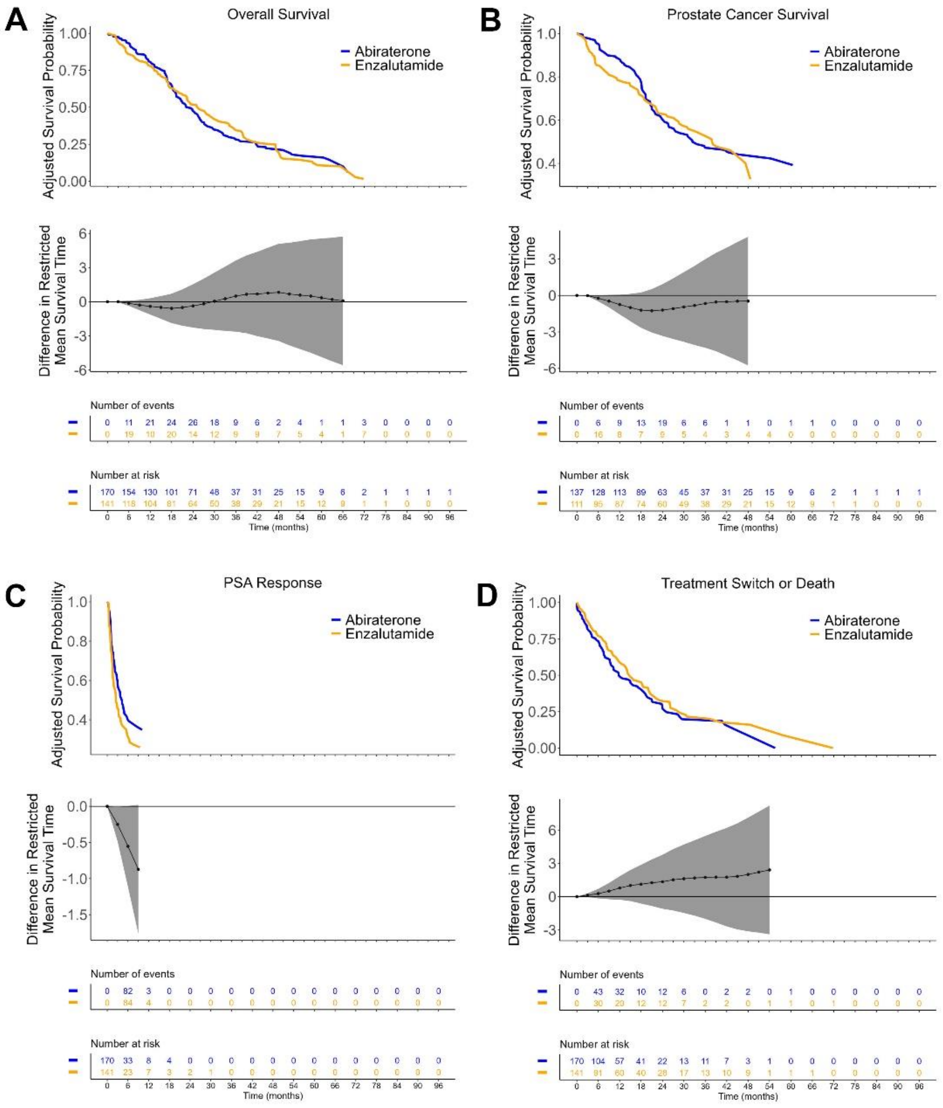

© 2024 La J et al. *JAMA Network Open*.

eFigure 19. Outcomes in Non-Hispanic Black patients treated with abiraterone acetate and enzalutamide therapy. In addition to Kaplan-Meier plots, the difference in restricted mean survival time (RMST) at each time point (three-month increments) is shown. The RMST at time *t* measures the mean survival censoring at *t*, and is equal to the area under the Kaplan-Meier plot up to *t*. The number of events and number at risk for abiraterone acetate (blue) or enzalutamide (orange) are tabulated under graphs at each timepoint. (A) Overall survival. (B) Prostate cancer survival. (C) Time to PSA response. (D) Time to treatment switch or death.

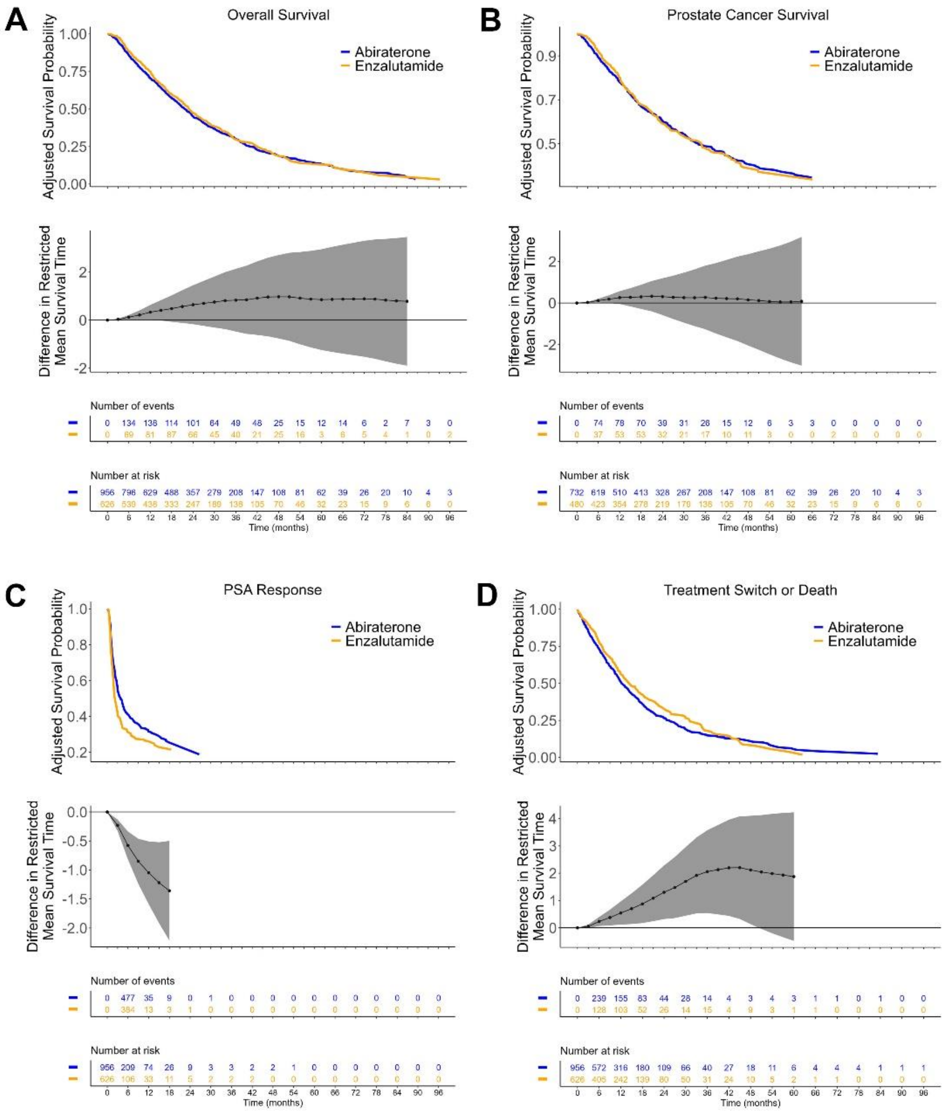

© 2024 La J et al. *JAMA Network Open*.

eFigure 20. Outcomes in Non-Hispanic White patients treated with abiraterone acetate and enzalutamide therapy. In addition to Kaplan-Meier plots, the difference in restricted mean survival time (RMST) at each time point (three-month increments) is shown. The RMST at time *t* measures the mean survival censoring at *t*, and is equal to the area under the Kaplan-Meier plot up to *t*. The number of events and number at risk for abiraterone acetate (blue) or enzalutamide (orange) are tabulated under graphs at each timepoint. (A) Overall survival. (B) Prostate cancer survival. (C) Time to PSA response. (D) Time to treatment switch or death.

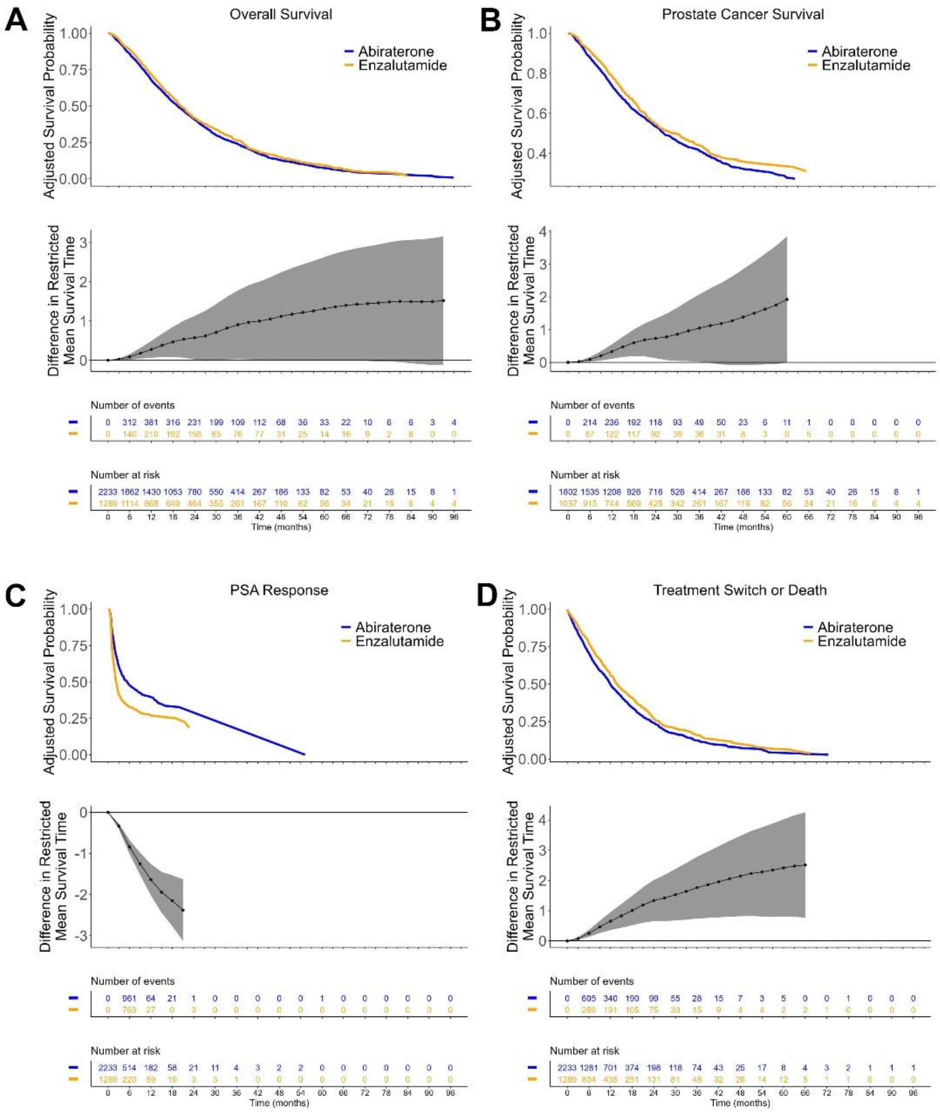

© 2024 La J et al. *JAMA Network Open*.

eFigure 21. Outcomes in patients 75 or older treated with abiraterone acetate and enzalutamide therapy. In addition to Kaplan-Meier plots, the difference in restricted mean survival time (RMST) at each time point (three-month increments) is shown. The RMST at time *t* measures the mean survival censoring at *t*, and is equal to the area under the Kaplan-Meier plot up to *t*. The number of events and number at risk for abiraterone acetate (blue) or enzalutamide (orange) are tabulated under graphs at each timepoint. (A) Overall survival. (B) Prostate cancer survival. (C) Time to PSA response. (D) Time to treatment switch or death.

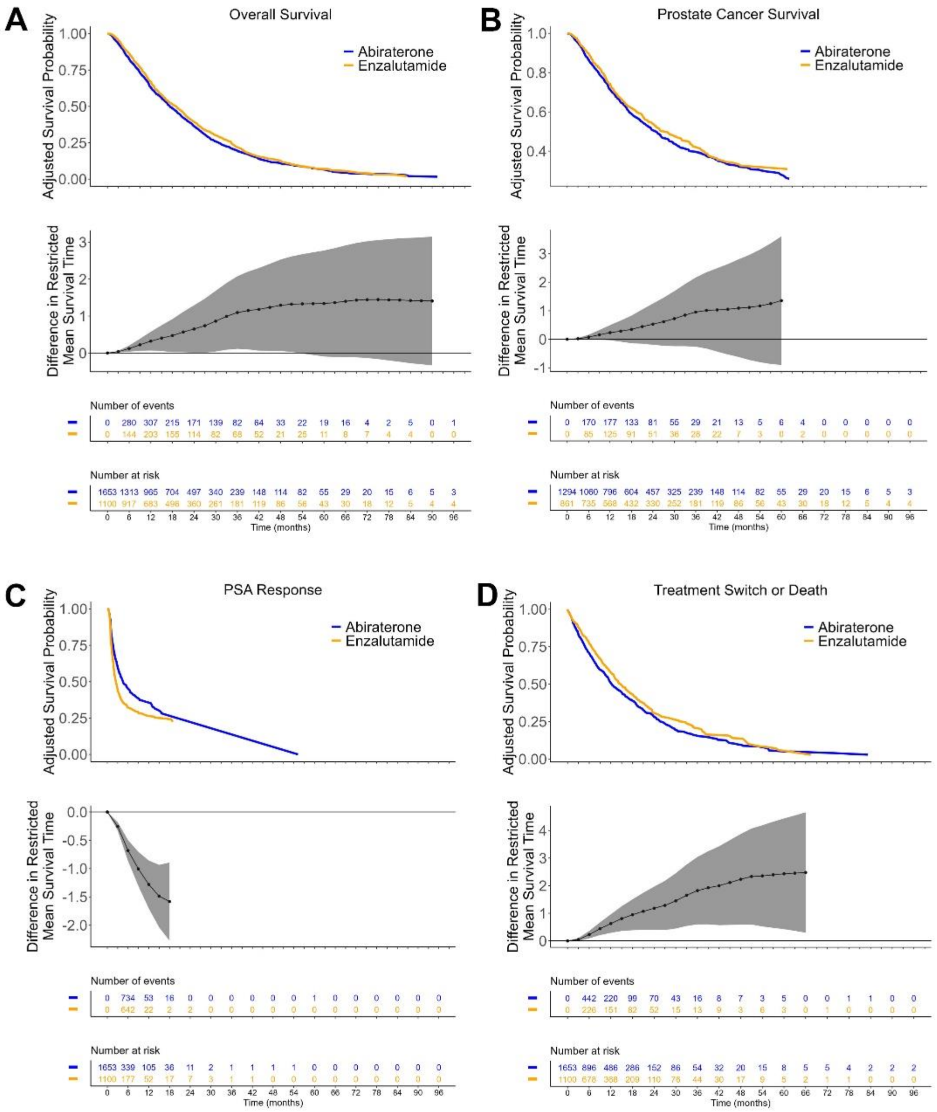

© 2024 La J et al. *JAMA Network Open*.

eFigure 22. Outcomes in patients younger than 75 treated with abiraterone acetate and enzalutamide therapy. In addition to Kaplan-Meier plots, the difference in restricted mean survival time (RMST) at each time point (three-month increments) is shown. The RMST at time *t* measures the mean survival censoring at *t*, and is equal to the area under the Kaplan-Meier plot up to *t*. The number of events and number at risk for abiraterone acetate (blue) or enzalutamide (orange) are tabulated under graphs at each timepoint. (A) Overall survival. (B) Prostate cancer survival. (C) Time to PSA response. (D) Time to treatment switch or death.

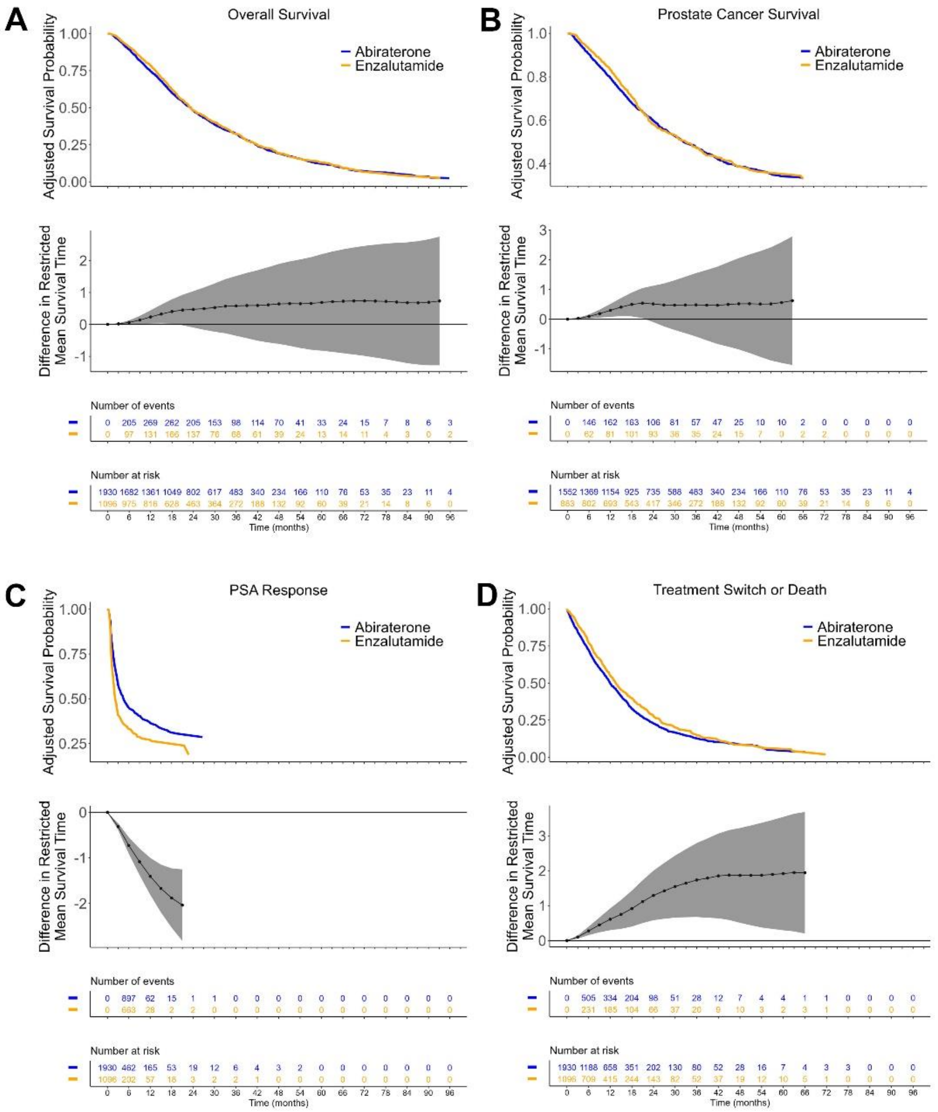

© 2024 La J et al. *JAMA Network Open*.
