# Supplementary material for: Abiraterone or Enzalutamide for Patients With Metastatic Castration-Resistant Prostate Cancer
Source: JAMA Netw Open. 2024 Aug 16;7(8):e2428444. doi: 10.1001/jamanetworkopen.2024.28444 (PMC11329885; doi:10.1001/jamanetworkopen.2024.28444)
Supplement: Supplement 2. — Data Sharing Statement [file jamanetwopen-e2428444-s002.pdf]

Data Sharing Statement

La. Abiraterone or Enzalutamide for Patients With Metastatic Castration-Resistant Prostate Cancer. *JAMA Netw Open*. Published August 16, 2024.
doi:10.1001/jamanetworkopen.2024.28444

Data
Data available: Yes
Data types: Data dictionary
How to access data: A data dictionary is included in the supplementary material.
When available: With publication

Supporting Documents
Document types: None

Additional Information
Who can access the data: The data dictionary will be available to all readers of the study in the supplementary material.
Types of analyses: The data dictionary will be available to support analyses for any purpose.
Mechanisms of data availability: The data dictionary will be freely available online in the study's supplementary material.
